# Three Versus 6 Months of Adjuvant Oxaliplatin-Fluoropyrimidine Chemotherapy for Colorectal Cancer: Final Results of SCOT—An International, Randomized, Phase III, Noninferiority Trial

**DOI:** 10.1200/JCO-25-00621

**Published:** 2026-01-09

**Authors:** Timothy Iveson, Mark P. Saunders, Caroline Kelly, Rachel S. Kerr, Jim Cassidy, Niels Henrik Hollander, Josep Tabernero, Andrew Haydon, Bengt Glimelius, Andrea Harkin, Karen Allan, John McQueen, Sarah Pearson, Kathleen A. Boyd, Andrew H. Briggs, Ashita Waterston, Louise Medley, Richard Ellis, Amandeep S. Dhadda, Mark Harrison, Stephen Falk, Charlotte Rees, Rene K. Olesen, David Propper, John Bridgewater, Ashraf Azzabi, David Cunningham, Tamas Hickish, Simon Gollins, Harpreet S. Wasan, David Church, Enric Domingo

**Affiliations:** ^1^ University of Southampton, Southampton, United Kingdom; ^2^ The Christie Hospital, Manchester, United Kingdom; ^3^ Glasgow Oncology Clinical Trials Unit, School of Cancer Sciences, University of Glasgow, Glasgow, United Kingdom; ^4^ Department of Oncology, University of Oxford, Oxford, United Kingdom; ^5^ Department of Oncology and Palliative Care, Zealand University Hospital, Naestved, Denmark; ^6^ Vall d'Hebron Hospital Campus and Institute of Oncology (VHIO), UVic-UCC, IOB-Quiron, CIBERONC, Barcelona, Spain; ^7^ Australasian Gastro-Intestinal Trials Group (AGITG), Alfred Hospital, Melbourne, Australia; ^8^ Department of Immunology, Genetics and Pathology, University of Uppsala, Uppsala, Sweden; ^9^ OCTO, Department of Oncology, University of Oxford, Oxford, United Kingdom; ^10^ Institute of Health and Wellbeing, University of Glasgow, Glasgow, United Kingdom; ^11^ Beatson West of Scotland Cancer Centre, NHS GGC HB, Glasgow, United Kingdom; ^12^ Royal United Hospital, Bath, United Kingdom; ^13^ Royal Cornwall Hospitals NHS Trust, Cornwall, United Kingdom; ^14^ Castle Hill Hospital, Hull, United Kingdom; ^15^ Mount Vernon Cancer Centre, Northwood, United Kingdom; ^16^ Bristol Cancer Institute, Bristol, United Kingdom; ^17^ Southampton University Hospital NHS Foundation Trust, Southampton, United Kingdom; ^18^ Department of Oncology, Aarhus University Hospital, Aarhus C, Denmark; ^19^ Barts Cancer Institute, Queen Mary, University of London, London, United Kingdom; ^20^ University College London, London, United Kingdom (part funded by the UCLH/UCL Biomedical Research Centre); ^21^ Newcastle upon Tyne Hospitals NHS Foundation Trust, Newcastle, United Kingdom; ^22^ Royal Marsden, London, United Kingdom (funded by NIHR BRC at the Royal Marsden); ^23^ University Hospitals Dorset/Bournemouth University, Bournemouth, United Kingdom; ^24^ North Wales Cancer Treatment Centre, Rhyl, United Kingdom; ^25^ Hammersmith Hospital, Imperial College London, London, United Kingdom

## Abstract

Adjuvant chemotherapy for colorectal cancer (CRC) with oxaliplatin and fluoropyrimidine was traditionally given for 6 months but is associated with cumulative peripheral neuropathy. The SCOT study (ISRCTN59757862) was an international, randomized, phase III, noninferiority trial investigating treatment reduction from 6 to 3 months. It originally reported noninferior disease-free survival with reduced toxicity and improved quality of life for 3 months of treatment in 6,088 patients. Here, we report overall survival (OS) with 38 months of additional follow-up. Patients with high-risk stage II and stage III CRC were assigned (1:1) to receive 3 or 6 months of either capecitabine and oxaliplatin (CAPOX) or infusional fluorouracil, leucovorin, and oxaliplatin (FOLFOX; bolus and infused fluorouracil with oxaliplatin) that were selected before random assignment. With a median of 113 months follow-up and 1,255 OS events, 5-year OS for 3 versus 6 months of treatment was 82.4% in both groups (hazard ratio, 0.96; 95% CI, 0.8 to 1.07), proving noninferiority of 3 months of treatment. Noninferiority of 3 months of treatment for OS was also shown in 1,087 patients with rectal cancer. The duration effect is regimen-dependent with noninferiority shown for CAPOX but not for FOLFOX. In summary, SCOT has shown noninferiority for OS with 3 months of adjuvant chemotherapy treatment, which should be recommended for most patients.

## INTRODUCTION

In colorectal cancer (CRC), 6 months of adjuvant chemotherapy with oxaliplatin and a fluoropyrimidine^1-3^ has been an international standard for over 20 years. Consensus guidelines recommend doublet chemotherapy for stage III colon cancer; its use for high-risk stage II disease is more controversial.^4^ In both cases, concerns have been raised about long-term oxaliplatin-induced neurotoxicity, which is duration-dependent. To address whether reduced treatment duration could mitigate such toxicity without compromising efficacy, the IDEA collaboration performed six trials, each of which compared 3 versus 6 months of oxaliplatin and fluoropyrimidine chemotherapy. Pooled analysis of overall survival (OS) across these trials supported the use of 3 months of adjuvant capecitabine and oxaliplatin (CAPOX) for most patients with stage III colon cancer.^5^ The SCOT trial was the largest of these studies and the only individual study to confirm noninferiority for 3 months of treatment.^6^ SCOT also included patients with high-risk stage II colon cancer and was the only study within the IDEA collaboration to include patients with rectal cancer. Here, we present the OS results with long-term follow-up for the total study population and within subgroups.

## METHODS

Full details of the SCOT trial were provided in the original report and study protocol.^6^ The study was approved by the West Glasgow Research Ethics Committee (REC reference No. 07/S0703/136); all participants provided written informed consent before enrollment. OS and disease-free survival (DFS) end points were defined as the time from random assignment to death from any cause (OS) and to relapse, new CRC, or death from any cause (DFS), respectively. Survival plots used the Kaplan-Meier (KM) method; hazard ratios (HRs) were calculated by multivariable Cox proportional hazards models using complete follow-up data and minimization factors of treatment regimen chosen by treating clinician (CAPOX or infusional fluorouracil, leucovorin, and oxaliplatin [FOLFOX]), sex, disease site (colon and rectum), T stage, and N stage as covariables. Proportionality of hazards was verified by scaled Schoenfeld residuals and R function cox.zph; restricted mean survival time models were run in cases of deviation. Tests for noninferiority used a HR of 1.13 for DFS and OS from Cox models as reported previously.^6^ Statistical tests were two-sided and significance accepted at *P* < .05.

## RESULTS

**FIG 1.**
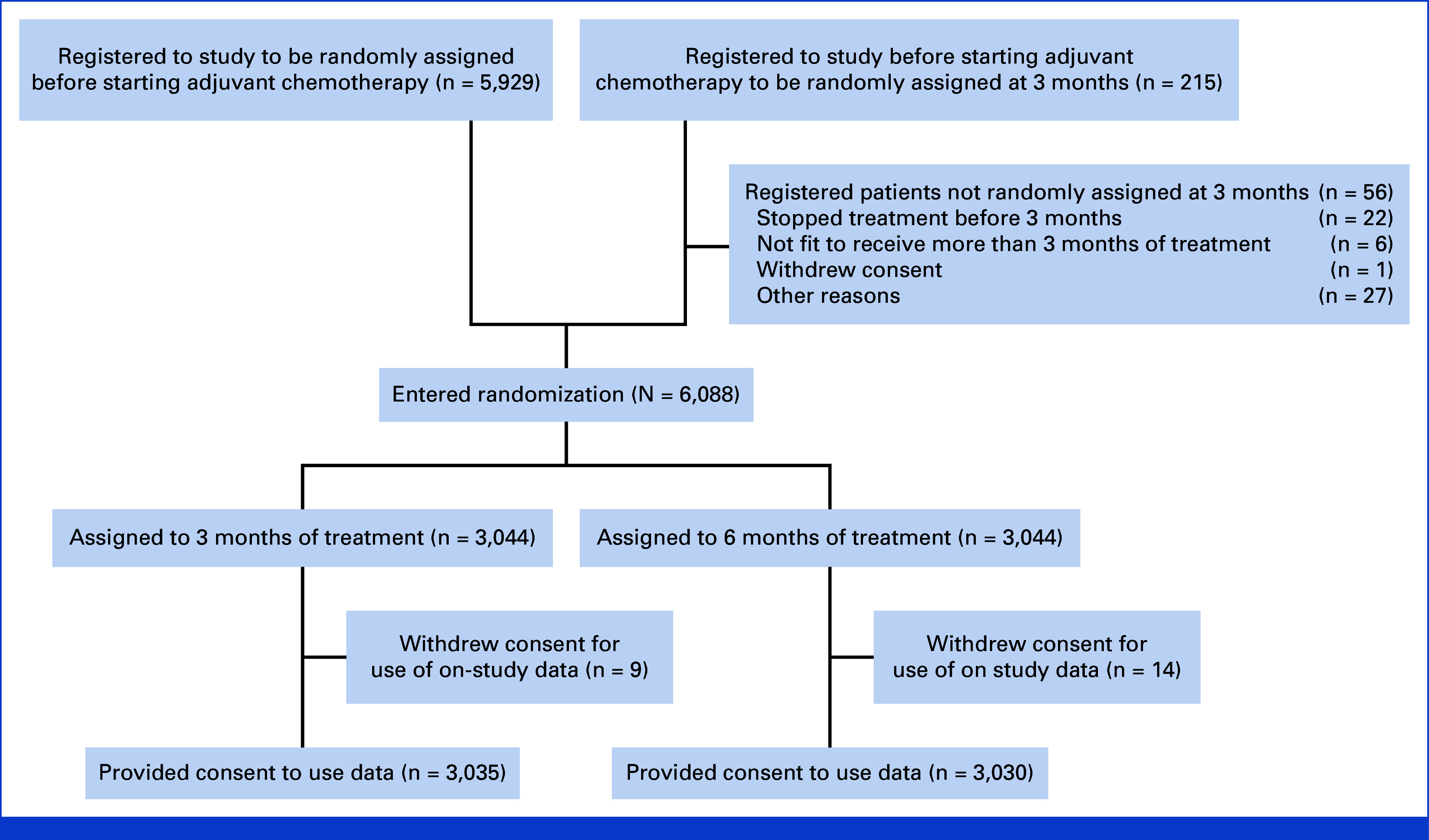
Study CONSORT diagram.

**FIG 2.**
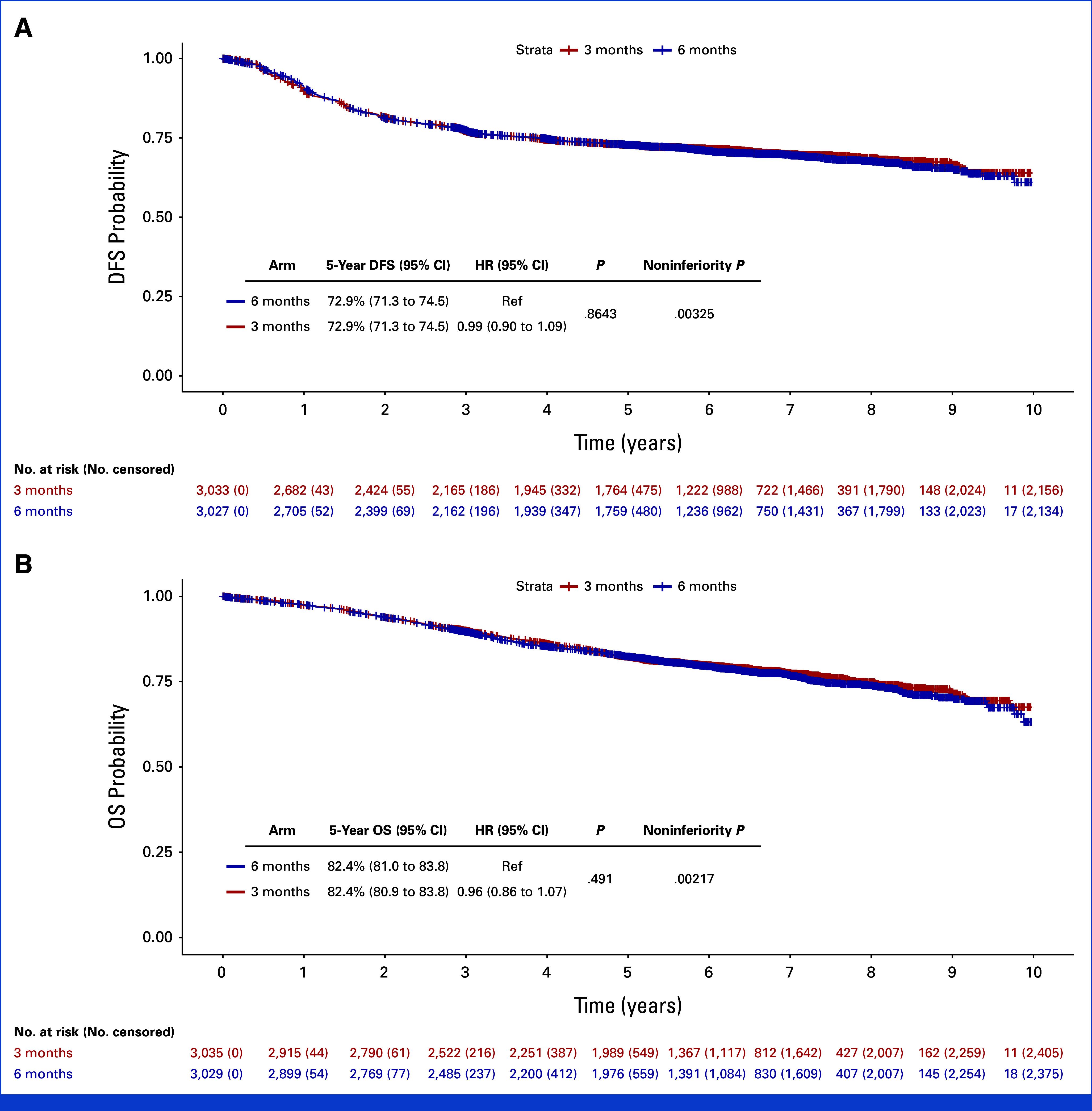
(A) DFS and (B) OS by duration of chemotherapy in the total study population. Kaplan-Meier curves showing probability of DFS according to chemotherapy duration. HR was calculated by Cox proportional hazards model using study minimization factors as covariables (see Methods for details). Noninferiority *P* was calculated by z-test. DFS, disease-free survival; HR, hazard ratio; OS, overall survival.

**FIG A1.**
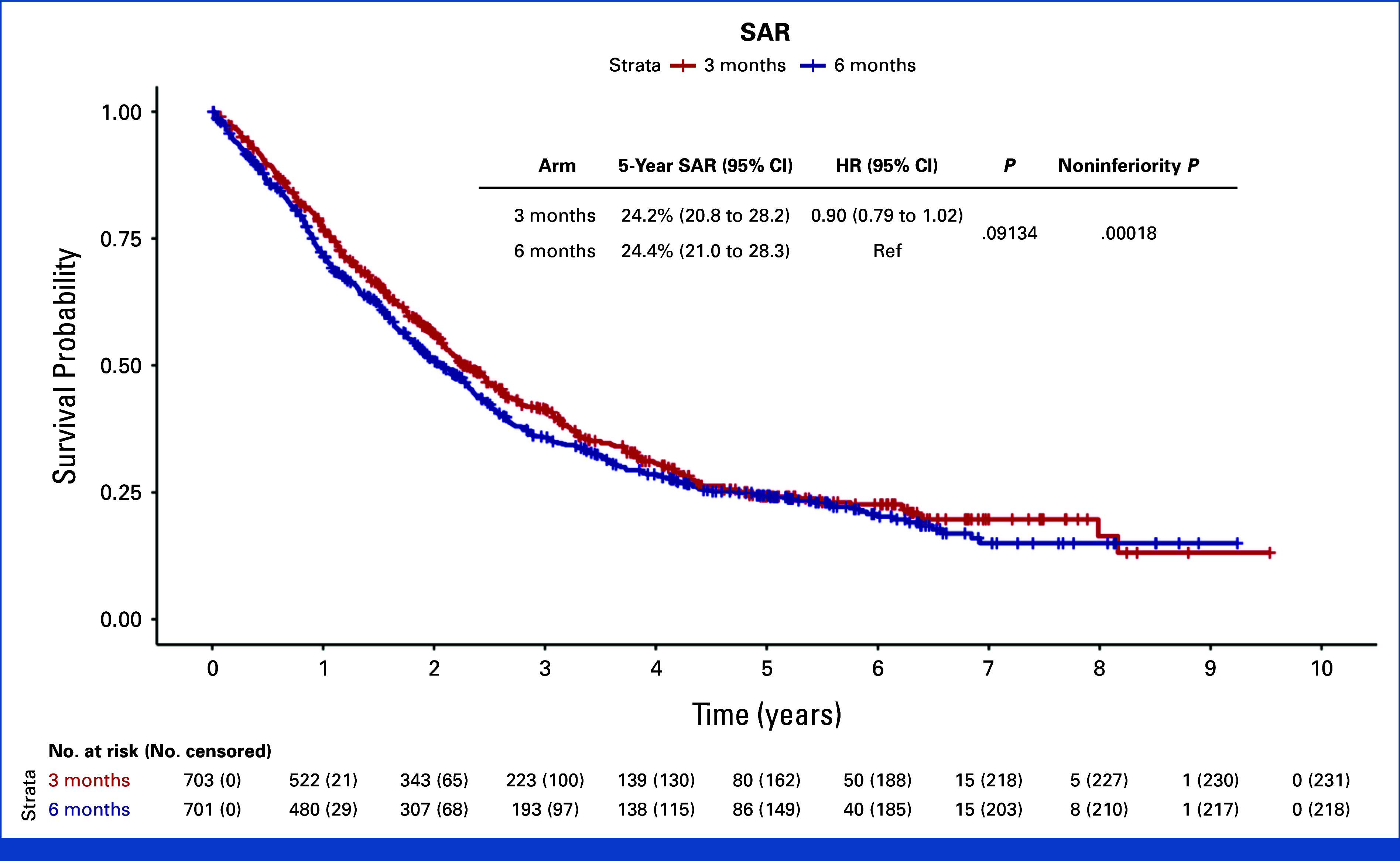
SAR by duration of adjuvant chemotherapy. Kaplan-Meier curves showing survival after colorectal cancer recurrence in patients with relapse from the total study population. HR was calculated by Cox proportional hazards model using study minimization factors as covariables (see Methods for details). Noninferiority *P* was calculated by using z-test. HR, hazard ratio; SAR, survival after recurrence.

A total of 6,088 patients were randomly assigned between March 27, 2008, and November 29, 2013, of whom 4,109 (67.5%) received CAPOX and 1,979 (32.5%) FOLFOX (Fig 1). Patient characteristics according to chemotherapy duration (Table 1) and regimen (Appendix Table A1, online only) were well balanced between groups, with the exception of slightly higher proportion of N1 disease in FOLFOX-treated cases. OS data were updated to November 30, 2019, resulting in median follow-up of 75 months (IQR, 61-91; reverse KM). Toxicity and quality-of-life data were not updated after the initial trial report. Final analysis of the study primary end point of DFS included 1,747 events and revealed a 5-year DFS of 72.9% for both 3 months and 6 months of treatment (HR, 0.99; 95% CI, 0.90 to 1.09; Fig 2A). Analysis of OS included 1,255 events and demonstrated a 5-year OS of 82.4% for both 3 months and 6 months of treatment (HR, 0.96; 95% CI, 0.86 to 1.07; Fig 2B), the upper margin of the 95% CI formally confirming noninferiority for 3 months of adjuvant treatment for OS (noninferiority *P* = .0033). Corresponding analysis of survival after relapse is provided in the supplementary material (Appendix Fig A1).

**TABLE 1.** Baseline Patient Characteristics by Chemotherapy Duration

Characteristic	3 Months (n = 3,044), No. (%)	6 Months (n = 3,044), No. (%)	*P* ^a^
Sex			>.9
Female	1,200 (39)	1,201 (39)	
Male	1,844 (61)	1,843 (61)	
Age, years, median (IQR)	65 (58-70)	65 (58-70)	.8
Performance status			.2
0	2,190 (72)	2,144 (70)	
1	854 (28)	900 (30)	
Disease site			>.9
Colon	2,492 (82)	2,495 (82)	
Rectum	552 (18)	549 (18)	
T stage			>.9
0	0 (0)	1 (<0.1)	
1	93 (3.1)	97 (3.2)	
2	281 (9.2)	280 (9.2)	
3	1,751 (58)	1,753 (58)	
4	918 (30)	913 (30)	
X	1 (<0.1)	0 (0)	
N stage			>.9
0	552 (18)	544 (18)	
1	1,737 (57)	1,740 (57)	
2	755 (25)	760 (25)	
Planned treatment			>.9
CAPOX	2,053 (67)	2,056 (68)	
FOLFOX	991 (33)	988 (32)	
Randomization time point			>.9
Baseline	2,964 (97)	2,965 (97)	
3 months	80 (2.6)	79 (2.6)	
High risk (T4 and/or N2)			.8
Low	1,615 (53)	1,626 (53)	
High	1,428 (47)	1,418 (47)	
Missing	1	0	

Abbreviation: CAPOX, capecitabine and oxaliplatin; FOLFOX, infusional fluorouracil, leucovorin, and oxaliplatin.

^a^ Pearson's chi-square test; Wilcoxon rank sum test; Fisher's exact test.

**FIG 3.**
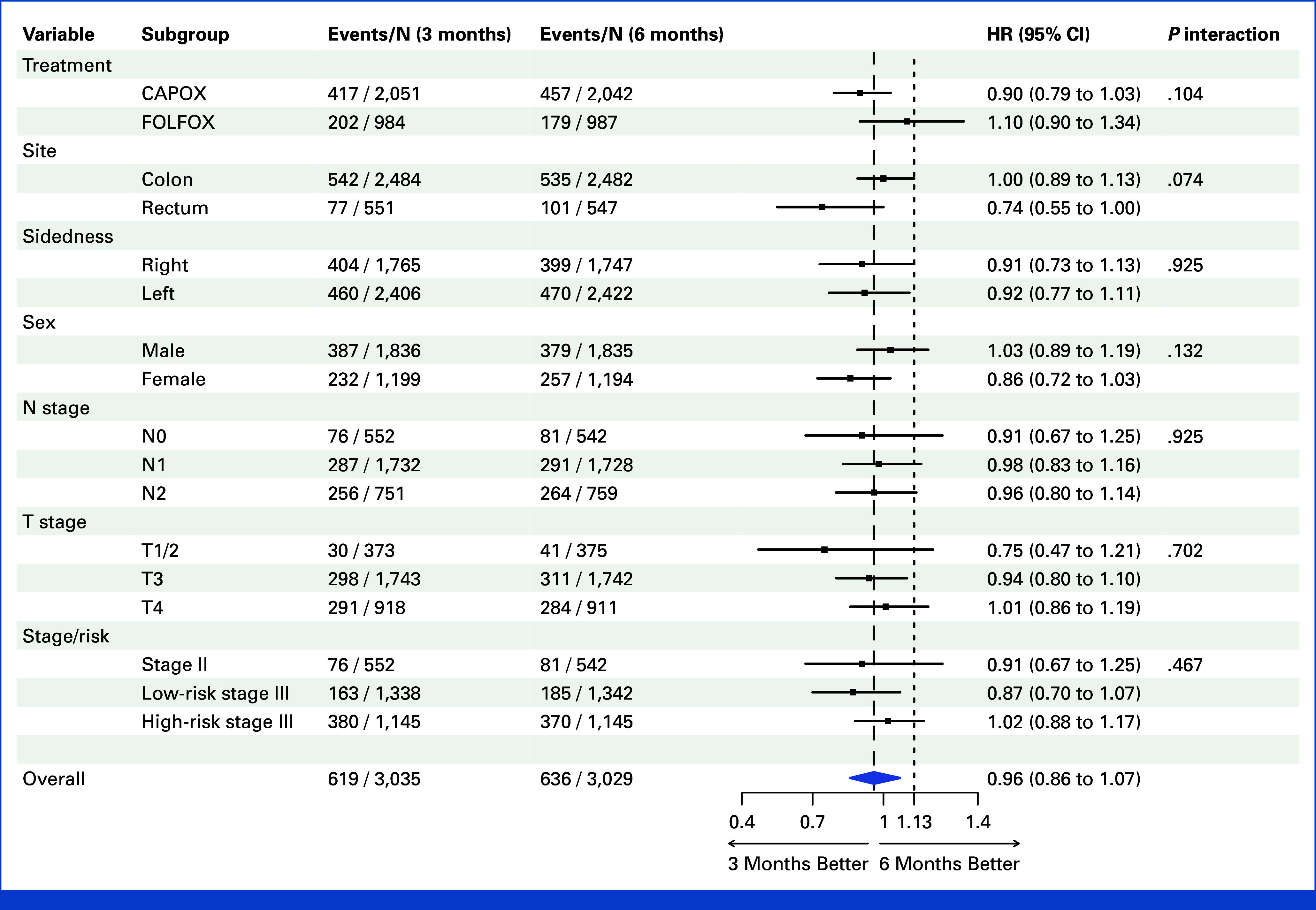
Overall survival by duration of chemotherapy within patient subgroups. Forest plot shows HR for overall survival with 3 months of chemotherapy relative to referent of 6 months of treatment. Proportionality of hazards between groups was not met for analysis of rectal cancers; corresponding analysis by restricted mean survival time is provided in Appendix Table A2. CAPOX, capecitabine and oxaliplatin; FOLFOX, infusional fluorouracil, leucovorin, and oxaliplatin; HR, hazard ratio.

**FIG A2.**
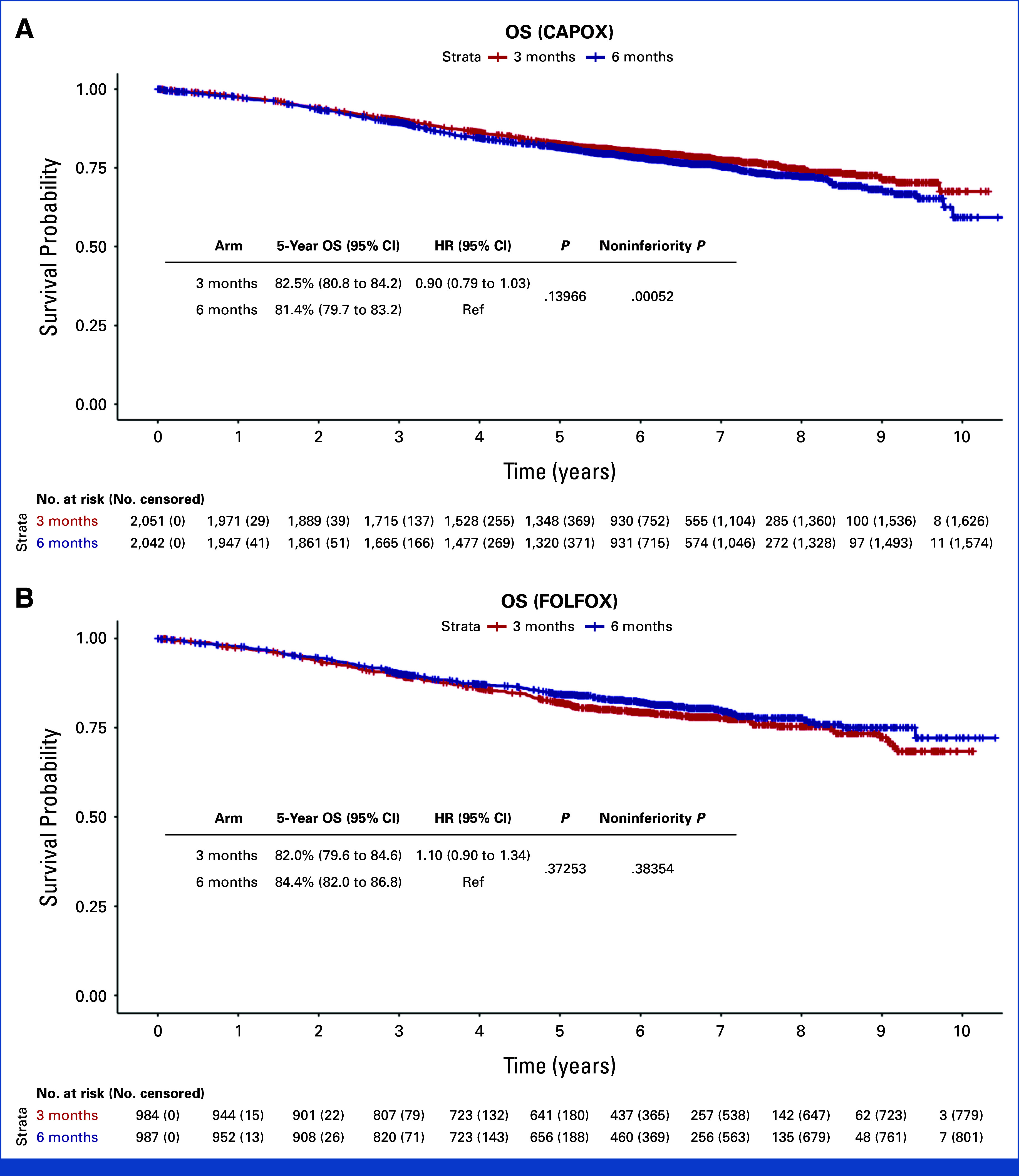
OS by duration of adjuvant chemotherapy according to treatment regimen. Kaplan-Meier curves showing OS by chemotherapy duration in patients treated with (A) CAPOX and (B) FOLFOX. HRs were calculated by Cox proportional hazards model using study minimization factors as covariables (see Methods for details). Noninferiority *P* was calculated by using z-test. CAPOX, capecitabine and oxaliplatin; FOLFOX, infusional fluorouracil, leucovorin, and oxaliplatin; HR, hazard ratio; OS, overall survival.

Previous secondary end point analysis revealed the effect of treatment duration depended on chemotherapy regimen.^5,6^ For patients treated with CAPOX, 5-year OS for 3 versus 6 months of treatment was 82.5% and 81.4%, respectively (HR, 0.90; 95% CI, 0.78 to 1.03; Fig 3, Appendix Fig A2). For FOLFOX, corresponding 5-year OS for 3 versus 6 months of treatment was 82.0% and 84.4%, respectively (HR, 1.10; 95% CI, 0.93 to 1.34; Fig 3, Appendix Fig A2). Thus, for OS, noninferiority of 3 months of treatment was confirmed for CAPOX (*P* = .00052) but not for FOLFOX (*P* = .38).

**FIG A3.**
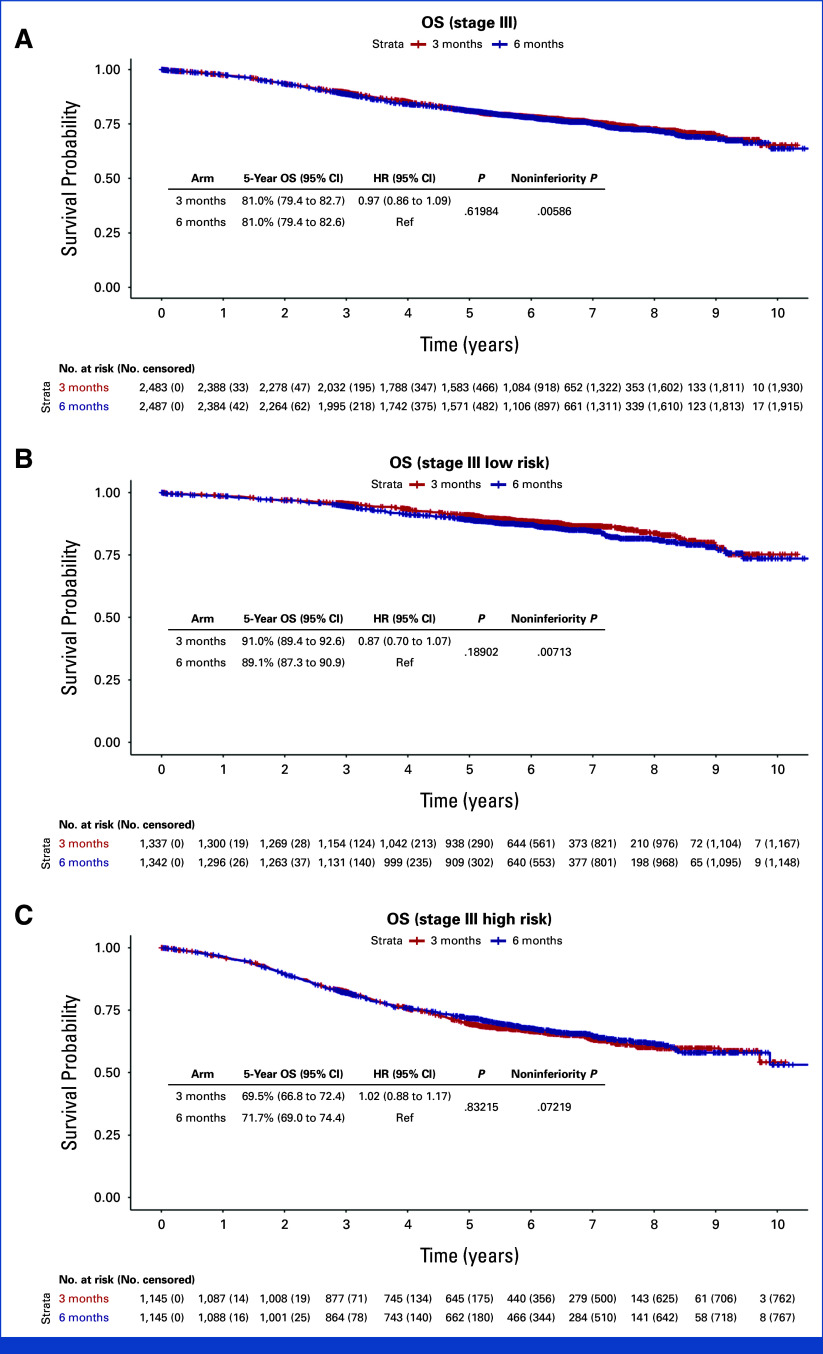
OS by duration of adjuvant chemotherapy in stage III disease overall and by risk group. Kaplan-Meier curves showing overall survival by chemotherapy duration in (A) all patients with stage III colorectal cancer and within (B) low-risk and (C) high-risk subgroups. HRs were calculated by Cox proportional hazards model using study minimization factors as covariables (see Methods for details). Noninferiority *P* was calculated by using z-test. HR, hazard ratio; OS, overall survival.

**FIG A4.**
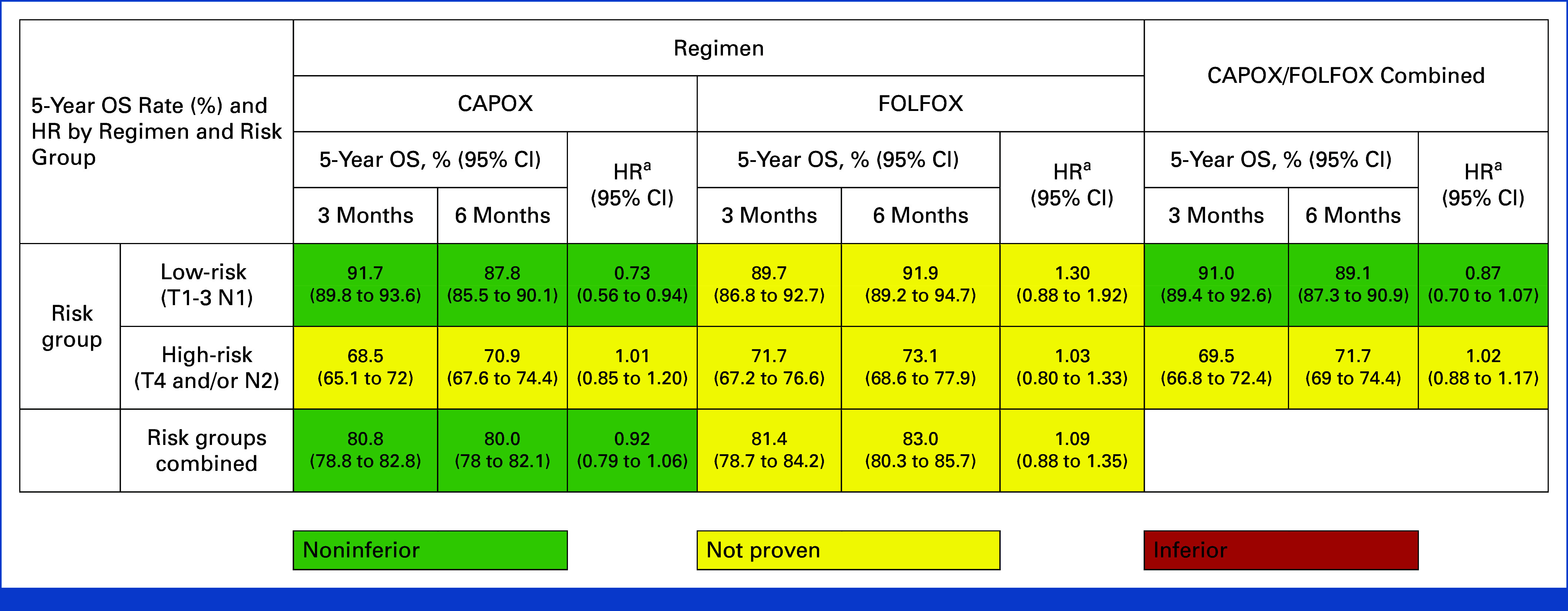
5-year OS and risk of death according to treatment duration in patients treated with CAPOX, FOLFOX, and across both groups. ^a^HR uses 6 months of chemotherapy treatment as referent. CAPOX, capecitabine and oxaliplatin; FOLFOX, infusional fluorouracil, leucovorin, and oxaliplatin; HR, hazard ratio; OS, overall survival.

Stage III colon cancers are often divided into low-risk (T3, N1) or high-risk (T4 and/or N2) disease for clinical management. OS for all stage III patients was 81.0% for both 3 and 6 months of treatment (Fig 3, Appendix Fig A3A). For low-risk cases, 5-year OS for 3 versus 6 months of treatment was 91.0% and 89.1%, respectively (HR, 0.87; 95% CI, 0.70 to 1.07; Fig 3, Appendix Table A1 and Fig A3B). For high-risk cases, 5-year OS for 3 versus 6 months of treatment was 69.5% and 71.7%, respectively (HR, 1.02; 95% CI, 0.88 to 1.17; Fig 3, Appendix Figs A3C and A4). In stage III disease, noninferiority of 3 months of chemotherapy for OS was therefore shown for low-risk (*P* = .0071) but not high-risk cases (*P* = .072; Fig 3, Appendix Figs A3 and A4).

**FIG A5.**
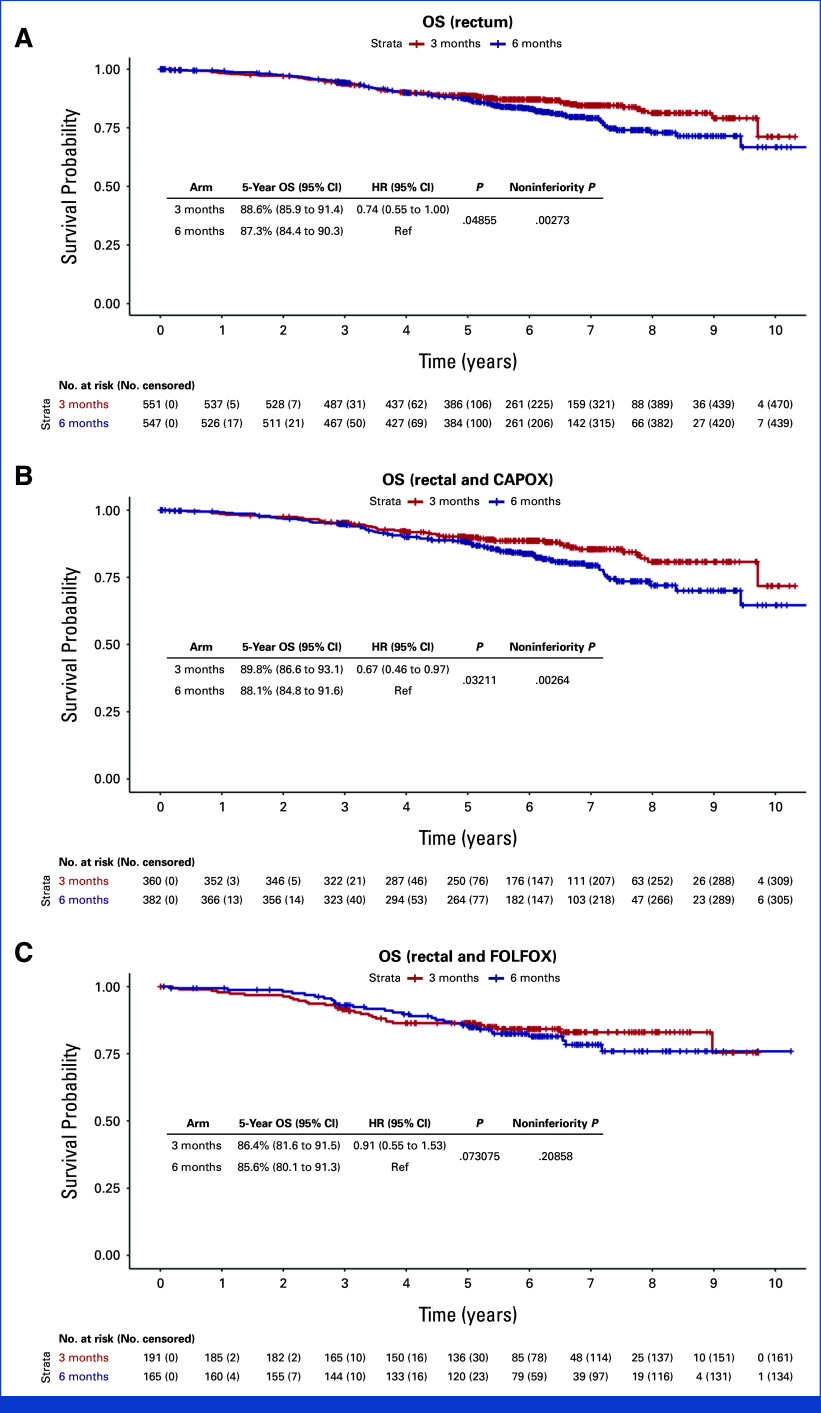
OS by duration of adjuvant chemotherapy in rectal cancer in all cases and by chemotherapy regimen. Kaplan-Meier curves showing overall survival by chemotherapy duration in (A) all patients with rectal cancers and in those treated with (B) CAPOX and (C) FOLFOX. HRs were calculated by Cox proportional hazards model using study minimization factors as covariables (see Methods for details). Analysis of scaled Schoenfeld residuals revealed nonproportionality of hazards; analysis by restricted mean survival time is shown in Appendix Figure A4. Noninferiority *P* was calculated by using z-test. CAPOX, capecitabine and oxaliplatin; FOLFOX, infusional fluorouracil, leucovorin, and oxaliplatin; HR, hazard ratio; OS, overall survival.

SCOT included 1,101 patients with rectal cancer, in whom OS for 3 versus 6 months of treatment was 88.6% and 87.3%, respectively (HR, 0.74; 95% CI, 0.55 to 1.00; Fig 3, Appendix Fig A5A). For rectal cancers treated with CAPOX, OS for 3 versus 6 months of treatment was 89.8% and 88.1%, respectively (HR, 0.67; 95% CI, 0.47 to 0.97; Appendix Fig A5B). For cases treated with FOLFOX, OS for 3 versus 6 months of treatment was 86.4% and 85.6%, respectively (HR, 0.91; 95% CI, 0.55 to 1.53; Appendix Fig A5C).

## DISCUSSION

Concerns about oxaliplatin-induced neurotoxicity motivated six studies to investigate whether adjuvant treatment for colon cancer could be shortened from 6 to 3 months. Their final pooled analysis was reported previously.^5^ Although noninferiority of 3 months of chemotherapy was not proven for the whole study population, prespecified analyses by regimen revealed differences, with noninferiority of attenuated treatment confirmed for CAPOX, but not for FOLFOX.^5^ SCOT was the largest of these studies and the only one to show noninferiority of 3 months of chemotherapy for the primary end point of DFS.^6^ This final analysis confirms noninferiority of 3 months of treatment for DFS with long-term follow up, and also demonstrates noninferiority for OS. The discordance with the previous pooled analysis likely reflects the fact that a substantially larger proportion of patients received CAPOX in the SCOT trial (67.5% *v* 39.5%).^5^

Among patients with stage III colon cancer, noninferiority of 3 months of chemotherapy for OS was proven for low-risk cases overall and those treated with CAPOX. Although noninferiority of attenuated treatment was not confirmed for patients with high-risk disease regardless of treatment regimen, or for patients treated with FOLFOX in either risk group, absolute differences in 5-year OS were modest (between 1.4% and 2.4%). The apparent variation in efficacy of attenuated chemotherapy by regimen was not obviously due to imbalance in prognostic factors between groups. It should, however, be noted these results are from analysis of secondary end points.

SCOT was the only study in the IDEA collaboration to include patients with rectal cancer, none of whom had received preoperative chemotherapy (short course radiotherapy was allowed). The demonstration of noninferiority for OS for 3 months of treatment is clinically relevant, as rectal cancers are increasingly treated with 3 months of preoperative chemotherapy as part of total neoadjuvant treatment. These results suggest that when such neoadjuvant chemotherapy is given, and particularly if CAPOX is used, further postoperative adjuvant treatment should be the exception rather than the rule.

These final SCOT results provide further evidence that 3 months of adjuvant chemotherapy can be recommended for most localized colon or rectal cancers, with no statistically significant OS advantage of 6 months of treatment in any cohort (Appendix Fig A4). Even for patients with high-risk stage III disease receiving FOLFOX, for whom the differences between 3 and 6 months of treatment were largest, absolute OS differences were small (2.2%), meaning that 50 patients would need to be treated with 6 months of treatment to prevent one death. This should be weighed against increased grade ≥2 peripheral neuropathy from longer treatment duration (from 25% to 58%), which translates to at least 15 patients having long-lasting clinically significant impairment.

When deciding on treatment duration for high-risk stage III disease, it is important to emphasize that there is no significant OS advantage for 6 months of therapy, and to highlight the additional toxicities (particularly cumulative neuropathy) and hospital visits. Extended chemotherapy increases pressure on delivery units and, combined with management of excess toxicities, increases financial burden. We contend that the substantial majority of patients with localized colon and rectal cancer should receive 3 months of adjuvant treatment. Extended treatment duration should be the exception, and only agreed after careful shared decision making between clinician and patient.

## Data Availability

A data sharing statement provided by the authors is available with this article at DOI https://doi.org/10.1200/JCO-25-00621.

## APPENDIX

**TABLE A1.** Baseline Patient Characteristics by Chemotherapy Regimen

Characteristic	SCOT, No. (%)		*P* ^a^
	CAPOX n = 4,109	FOLFOX n = 1,979	
Arm, weeks			>.900
12	2,053 (50)	991 (50)	
24	2,056 (50)	988 (50)	
Sex			.500
Female	1,632 (40)	769 (39)	
Male	2,477 (60)	1,210 (61)	
Age, years, median (Q1, Q3)	65 (58, 70)	65 (59, 70)	.200
Performance status			.093
0	2,953 (72)	1,381 (70)	
1	1,156 (28)	598 (30)	
Disease site			.900
Colon	3,364 (82)	1,623 (82)	
Rectum	745 (18)	356 (18)	
Sidedness			.600
Right	860 (32)	376 (33)	
Left	1,798 (68)	757 (67)	
Missing	1,451	846	
T stage			.500
0	0 (0)	1 (<0.1)	
1	123 (3.0)	67 (3.4)	
2	367 (8.9)	194 (9.8)	
3	2,381 (58)	1,123 (57)	
4	1,237 (30)	594 (30)	
X	1 (<0.1)	0 (0)	
N stage			.002
0	789 (19)	307 (16)	
1	2,309 (56)	1,168 (59)	
2	1,011 (25)	504 (25)	
High risk (T4 and/or N2)			.500
Low	2,199 (54)	1,042 (53)	
High	1,909 (46)	937 (47)	
Missing	1	0	

Abbreviations: CAPOX, capecitabine and oxaliplatin; FOLFOX, infusional fluorouracil, leucovorin, and oxaliplatin.

^a^ Pearson's chi-square test; Wilcoxon rank sum test; Fisher's exact test.

**TABLE A2.** RMST Analysis of Rectal Cancers

RMST					
Subset	Arm	Cutoff	Est	Ratio (95% CI)	*P*
Rectum	3 months	9.72	8.69	0.96 (0.93 to 1.00)	.051
	6 months	9.72	8.37		
Rectum and CAPOX	3 months	9.72	8.78	0.95 (0.91 to 1.00)	.031
	6 months	9.72	8.36		
Rectum and FOLFOX	3 months	9.72	8.54	0.99 (0.92 to 1.06)	.740
	6 months	9.72	8.44		

Abbreviations: CAPOX, capecitabine and oxaliplatin; FOLFOX, infusional fluorouracil, leucovorin, and oxaliplatin; RMST, restricted mean survival time.

## References

[1] Andre T Boni C Mounedji-Boudiaf L et al Oxaliplatin, fluorouracil, and leucovorin as adjuvant treatment for colon cancer N Engl J Med 350 2343 2351 2004 15175436 10.1056/NEJMoa032709

[2] Kuebler JP Wieand HS O'Connell MJ et al Oxaliplatin combined with weekly bolus fluorouracil and leucovorin as surgical adjuvant chemotherapy for stage II and III colon cancer: Results from NSABP C-07 J Clin Oncol 25 2198 2204 2007 17470851 10.1200/JCO.2006.08.2974

[3] Haller DG Tabernero J Maroun J et al Capecitabine plus oxaliplatin compared with fluorouracil and folinic acid as adjuvant therapy for stage III colon cancer J Clin Oncol 29 1465 1471 2011 21383294 10.1200/JCO.2010.33.6297

[4] Chibaudel B Raeisi M Cohen R et al Assessment of the addition of oxaliplatin to fluoropyrimidine-based adjuvant chemotherapy in patients with high-risk stage II Colon cancer: An ACCENT pooled analysis J Clin Oncol 42 4187 4195 2024 39231393 10.1200/JCO.24.00394 PMC11624096

[5] Andre T Meyerhardt J Iveson T et al Effect of duration of adjuvant chemotherapy for patients with stage III colon cancer (IDEA collaboration): Final results from a prospective, pooled analysis of six randomised, phase 3 trials Lancet Oncol 21 1620 1629 2020 33271092 10.1016/S1470-2045(20)30527-1 PMC7786835

[6] Iveson TJ Kerr RS Saunders MP et al 3 versus 6 months of adjuvant oxaliplatin-fluoropyrimidine combination therapy for colorectal cancer (SCOT): An international, randomised, phase 3, non-inferiority trial Lancet Oncol 19 562 578 2018 29611518 10.1016/S1470-2045(18)30093-7 PMC5883334

