# Supplementary material for: Three Versus 6 Months of Adjuvant Oxaliplatin-Fluoropyrimidine Chemotherapy for Colorectal Cancer: Final Results of SCOT—An International, Randomized, Phase III, Noninferiority Trial
Source: J Clin Oncol. 2026 Jan 9;44(7):534–9. doi: 10.1200/JCO-25-00621 (PMC12931879; doi:10.1200/JCO-25-00621)
Supplement: Supplementary file 2 [file jco-44-534-s002.pdf]

SCOT STUDY SCOT – Short Course Oncology Therapy – A Study of Adjuvant Chemotherapy in Colorectal Cancer*

* Incorporating the Extension of Follow-up for High Risk Stage II patients (additional 3 years) and Stage III Patients (up to year 3 follow-up) (HTA Project Reference 14/140/84)

Version:	Version 8.0
Date:	20th December 2017
EudraCT number:	2007-003957-10
ISRCTN number:	ISRCTN59757862

This study is jointly sponsored by The University of Glasgow and Greater Glasgow Health Board

PROTOCOL APPROVAL

SCOT – Short Course Oncology Therapy – A Study of Adjuvant Chemotherapy in Colorectal Cancer

Chief Investigator: **Dr Tim Iveson**

Southampton University Hospitals NHS Trust
Tremona Road
Southampton
SO16 6YD

Signature:

Date:

Participating Site

Site Name: ____

Principal Investigator at Site: ____

Signature: ____

Date: ____

SCOT Trial Management Group

Chief Investigator

Dr Tim Iveson, Southampton University Hospitals NHS Trust
Tel: +44(0) 2381 206802 Fax: +44(0) 2381 295176
E-mail: mailto:tim.iveson@uhs.nhs.uk

Deputy Chief Investigator and Lead Clinician for Translational Research

Dr Rachel Kerr, University of Oxford
Tel: +44(0) 1865 617056 Fax: +44(0) 1865 617010
E-mail: rachel.kerr@oncology.ox.ac.uk

Co-Investigator

Dr Mark Saunders, The Christie Hospital, Manchester
Tel: +44(0) 161 446 3357 Fax: +44(0) 161 446 3648
E-mail: Mark.Saunders@christie.nhs.uk

Trial Statistician

Mr Jim Paul, Cancer Research UK Clinical Trials Unit
Tel: +44(0) 141 301 7188 Fax: +44(0) 141 301 7189
E-mail: james.paul@glasgow.ac.uk

Health Economics

Professor Andrew Briggs, University of Glasgow
Tel: +44(0) 141 330 5017 Fax: +44(0) 141 330 5018
E-mail: andrew.briggs@glasgow.ac.uk

Project Management

Mrs Andrea Harkin, Cancer Research UK Clinical Trials Unit
Tel: +44(0) 141 301 7186 Fax: +44(0) 141 301 7192
E-mail: andrea.harkin@glasgow.ac.uk

Pharmacovigilance/Quality Assurance

Mrs Lindsey Connery (Lead PV Contact), Cancer Research UK Clinical Trials Unit
Tel: +44(0) 141 301 7209 Fax: +44(0) 141 301 7213
E-mail: lindsey.connery@glasgow.ac.uk

Clinical Trial Monitors

Ms Michaela Rodger, Cancer Research UK Clinical Trials Unit
Tel: +44(0) 141 301 7941 Fax: +44(0) 141 301 7187
E-mail: michaela.rodger@glasgow.ac.uk

Clinical Trial Co-ordinators/Administrators

Mr John McQueen, Cancer Research UK Clinical Trials Unit
Tel: +44(0) 141 301 7191 Fax: +44(0) 141 301 7192
E-mail: john.mcqueen@glasgow.ac.uk

Postal Addresses for Study Contacts

Cancer Research UK Clinical Trials Unit
Level 0
The Beatson West of Scotland Cancer Centre
1053 Great Western Road
GLASGOW
G12 0YN

University of Glasgow
University Avenue
GLASGOW
G12 1QQ

Southampton University Hospitals NHS Trust
Tremona Road
SOUTHAMPTON
SO16 6YD

Sponsor

The Sponsor of this clinical trial is Greater Glasgow Health Board/University of Glasgow (GU).

Contact:
Joanne McGarry
Academic Research Co-ordinator
Greater Glasgow Health Board
Research and Development Management Office
West Glasgow Ambulatory Care Hospital
Dalnair Street
Glasgow
G3 8SW
Tel: 0141 232 1818
E-mail: joanne.mcgarry@ggc.scot.nhs.uk

Funder

Medical Research Council (MRC)
14th Floor
One Kemble Street
London
WC2B 4AN

and

Medical Research Council (MRC)
2nd Floor David Phillips Building
Polari House
North Star Avenue
Swindon
Wiltshire
SN2 1FL

The Extension of Funding project was funded by the National Institute for Health Research HTA (project number 14/140/84).

The views and opinions expressed therein are those of the authors and do not necessarily reflect those of the HTA, NIHR, NHS or the Department of Health.

Study Summary

**STUDY TITLE:**	**SCOT**- **S**hort **C**ourse **O**ncology **T**herapy<br><br>A study of adjuvant chemotherapy in colorectal cancer.
**STUDY DESIGN:**	Phase III, randomised controlled, two arm, open label, multi-centre, non-inferiority.
**STUDY OBJECTIVES:**	Assessment of the efficacy of 12 weeks versus 24 weeks of treatment and associated toxicity.<br><br>Economic analysis of the cost effectiveness of the 2 arms.<br><br>Comparison of 2 randomisation methodologies.
**STUDY ENDPOINTS**	Primary Endpoint:<br>• Disease free survival<br><br>Secondary Endpoints:<br>• Overall survival<br>• Cost-effectiveness<br>• Toxicity<br>• Quality of Life
**STUDY POPULATION:**	The study randomised 6087 patients with fully resected high-risk stage II or fully resected stage III colorectal cancer before closing to recruitment on the 29th November 2013.
**RANDOMISATION:**	**The study is no longer recruiting patients**<br><br>To randomise a patient on to the study, contact the Cancer Research UK Clinical Trials Unit, Glasgow. Randomisation to the study can be done by either telephone or fax on the following numbers:<br><br>**Cancer Research UK Clinical Trials Unit, Glasgow**<br>**Telephone Number: 0141 301 7195**<br>**Fax Number: 0141 301 7192**<br><br>**From Quarter 2-3 of 2012 (21st August 2012)** all participating sites in the SCOT trial must contact their coordinating trials office (OCTO or CR-UK CTU Glasgow as appropriate) directly to randomise patients to the SCOT trial. CR-UK CTU Glasgow randomisation contact details will remain as per the above and those sites coordinated by OCTO should contact OCTO directly to randomise on the following numbers (please note that prior to **Quarter 2-3 of 2012 (21st August 2012)**, all sites should continue to contact CR-UK CTU Glasgow to randomise patients):<br><br>**Oncology Clinical Trials Office (OCTO), Oxford**<br>**UK Telephone Number 0800 389 1635**<br>**UK Fax Number 0800 389 1629**<br>**Non – UK Telephone Number:+44(0)1865617 014**<br>**Non – UK Fax Number:+44(0)1865 617 015**<br><br>During the first year of study recruitment, some sites were allocated to randomise patients after completion of 12 weeks

	of therapy (delayed randomisation). This was discontinued in July 2009, and all sites now randomise their patient prior to start of any adjuvant protocol treatment (please see Section 3 for full details).
**STUDY TREATMENT:**	Patients will be randomised to receive either 12 weeks or 24 weeks of adjuvant chemotherapy. The treatment regimen will be either oxaliplatin/5FU (OxMdG) or oxaliplatin/capecitabine (XELOX). Clinicians participating will be able to select which regimen they wish to use for each individual patient prior to randomisation.
**DURATION:**	Treatment duration is either 12 weeks or 24 weeks depending which arm is drawn. Patients will be followed up for a maximum of 9 years.

SCHEDULE OF ASSESSMENTS

	Screening			Treatment Cycle												Follow Up							
**Study Procedures**	**≤28 days prior to Reg/Rand**	**≤7 days prior to Reg/Rand**	**Reg/Rand**	**Cycle 1 (Day1)**	**Cycle 2 (Day1)**	**Cycle 3 (Day1)**	**Cycle 4 (Day1)**	**Cycle 5 (Day1)**	**Cycle 6 (Day1)**	**cycle 7 (Day1)**	**Cycle 8 (Day1)**	**Cycle 9 (Day1)**	**Cycle 10 (Day1)**	**Cycle 11 (Day1)**	**Cycle 12 (Day1)**	**Mth 4 & 5 Post reg/rand(2)**	**Mth 6 post reg/rand**	**Mth 9 post reg/rand**	**Mth 12 post reg/rand**	**Mth 18 post reg/rand**	**Mth 24 post reg/rand**	**Mth 36 post reg/rand**	**(22) Annually until 9 yrs post reg/rand**
Informed Consent (1)	✓																						
Review of Eligibility Criteria		✓																					
Medical History		✓																					
Physical/Clinical Assessment (inc height and weight)		✓	Physical/Clinical Assessment post screening to be performed as per local standard practice																				
Body Surface Area		✓																					
ECG(3)		✓																					
WHO Performance Status		✓		✓	✓	✓	✓	✓	✓	✓	✓	✓	✓	✓	✓								
Toxicity Assessment				✓	✓	✓	✓	✓	✓	✓	✓	✓	✓	✓	✓	✓	✓						
Surgery(4)	To be within 13 weeks of cycle 1 date																						
Visualisation of Entire Colon (5)	Preferably within 3 months prior to or 3 months post surgery. Up to a maximum of 6 months pre surgery and a maximum of 12 months post surgery are acceptable																						
**Laboratory Procedures**																							
Haematology (Clotting (6))		✓(7)		✓(8)	✓(8)	✓(8)	✓(8)	✓(8)	✓(8)	✓(8)	✓(8)	✓(8)	✓(8)	✓(8)	✓(8)								
Haematology (FBC)		✓(7)		✓(8)	✓(8)	✓(8)	✓(8)	✓(8)	✓(8)	✓(8)	✓(8)	✓(8)	✓(8)	✓(8)	✓(8)	✓(8)	✓(8)	✓(8)	✓(8)	✓(8)	✓(8)	✓(8)	✓(8)
Urea & Electrolytes		✓(7)		✓(8)	✓(8)	✓(8)	✓(8)	✓(8)	✓(8)	✓(8)	✓(8)	✓(8)	✓(8)	✓(8)	✓(8)	✓(8)	✓(8)	✓(8)	✓(8)	✓(8)	✓(8)	✓(8)	✓(8)
Liver Function Tests (9)		✓(7)		✓(8)	✓(8)	✓(8)	✓(8)	✓(8)	✓(8)	✓(8)	✓(8)	✓(8)	✓(8)	✓(8)	✓(8)	✓(8)	✓(8)	✓(8)	✓(8)	✓(8)	✓(8)	✓(8)	✓(8)
CEA – XELOX Regimen(10) (11)		✓(7)			✓(8)		✓(8)		✓(8)		✓(8)					✓(8)	✓(8)	✓(8)	✓(8)	✓(8)	✓(8)	✓(8)	✓(8)
CEA – OxMdG Regimen(10) (12)		✓(7)				✓(8)			✓(8)			✓(8)			✓(8)	✓(8)	✓(8)	✓(8)	✓(8)	✓(8)	✓(8)	✓(8)	✓(8)
Urine Pregnancy Test		✓(7)																					
**Radiological Assessment**																							
CT Scan (chest, abdomen and pelvis) (13)	To be within 16 weeks pre-study entry max allowed 16 weeks 5 days																✓(14)		✓(14)	✓(14)	✓(14)	✓(14)	
**Patient Questionnaires**																							
EORTC QLQ-C30 & CR29 QoL(15) (16) (18)		✓(16)			✓	✓	✓	✓	✓	✓	✓	✓	✓	✓	✓	✓	✓(15)	✓	✓				
EQ-5D (15) (16) (18)		✓(16)			✓	✓	✓	✓	✓	✓	✓	✓	✓	✓	✓	✓	✓(15)	✓	✓	✓	✓	✓	✓
GOG Ntx 4 (15) (17) (18)		✓(17)			✓	✓	✓	✓	✓	✓	✓	✓	✓	✓	✓	✓	✓(15)	✓	✓	✓	✓	✓	✓
Patient Withdrawal Questionnaire	Please ask the patient to complete this questionnaire if they stop their allocated treatment early **and/or** withdraw their consent from any aspect of the trial. If the patient is not willing to complete the questionnaire please record this on the Patient Withdrawal log. Post original questionnaire to OCTO/CTU Glasgow within 1 month of completion.																						

	Screening			Treatment Cycle												Follow Up							
	≤28 days prior to Reg/Rand	≤7 days prior to Reg/Rand	Reg/Rand	Cycle 1 (Day1) (4a)	Cycle 2 (Day1)	Cycle 3 (Day1)	Cycle 4 (Day1)	Cycle 5 (Day1)	Cycle 6 (Day1)	cycle 7 (Day1)	Cycle 8 (Day1)	Cycle 9 (Day1)	Cycle 10 (Day1)	Cycle 11 (Day1)	Cycle 12 (Day1)	Mth 4 & 5 Post reg/rand (2)	Mth 6 post reg/rand	Mth 9 post reg/rand	Mth 12 post reg/rand	Mth 18 post reg/rand	Mth 24 post reg/rand	Mth 36 post reg/rand	(22) Annually until 9 yrs post reg/rand
**CRFs/Forms**																							
Patient Consent Notification Form (18)			✓																				
Patient Registration/Randomisation Form (18)			✓																				
Treatment Forms (18)				✓	✓	✓	✓	✓	✓	✓	✓	✓	✓	✓	✓								
Follow up Forms (18)																✓	✓	✓	✓	✓	✓	✓	✓
Patient Consent Withdrawal Form	Please complete only if patient withdraws consent from any aspect of the trial and does NOT wish to have any further information included in the study, (from the date of withdrawal).<br>Please note this form should only be completed if the 'Survival Status' on the Follow Up CRF is checked as 'Patient has withdrawn consent'.<br>Site staff to please complete a 'Patient Consent Withdrawal Notification Form' and post original to OCTO/CTU Glasgow <u>within 1 month of completion.</u>																						
Patient Consent Withdrawal Notification Form	Please complete once patient has completed Consent Withdrawal Form and post original to OCTO/CTU Glasgow <u>within 1 month of completion.</u>																						
Pregnancy Notification	Please complete upon awareness of pregnancy of trial patient or pregnancy of trial patient's partner, fax immediately to OCTO/CTU Glasgow and post original to OCTO/CTU Glasgow <u>within 1 month of completion. Pregnancy should be reported during treatment and up to 1 year after last administration of study treatment. See protocol section 8.2 for full details.</u>																						
SAE Form				Please complete when a SAE occurs and fax to CTU Glasgow (fax # 0141 301 7213) within 24 hours of becoming aware of the event. SAEs should be reported up to 30 days after last administration of study treatment. See protocol section 7 for full details.																			
High Risk Stage II Patients Additional Information (19)				Please complete for patients identified at Randomisation as having high risk stage II disease (T3 N0 or T4 N0) and post original to OCTO/CTU Glasgow <u>within 1 month of completion.</u>																			
**transSCOT Translational Sub-Study**																							
Tissue collection (20)			Only a requirement for patients who have consented to collection of FFPE tumour tissue blocks																				
Blood Sample collection (21)			Only a requirement for patients who have consented to collection of additional blood samples (serum and EDTA)																				

(1) <u>All</u> patients must be consented to the study prior to randomisation and prior to any treatment starting.
(2) Only patients who are randomised to 12 weeks of treatment will attend for follow up at Month 4 and 5. After this point patients on both treatment arms are seen for follow up at the same time-points.
(3) A baseline ECG should be performed within 7 days of randomisation, however ECGs performed up to 20 days prior to randomisation will be accepted.
(4) If surgery to cycle 1 treatment start date is ≤ 13 weeks the patient will be considered eligible. However, randomization date to cycle 1 date must not exceed14 days. Please contact your co-ordinating trials office for clarification.
(4a) Cycle 1 treatment is to start within 14 days of randomization date.
(5) For example, by complete colonoscopy or CT virtual colonoscopy.
(6) Clotting/INR only required to be performed in patients on treatment with anti-coagulants.
(7) Pre-randomisation and C1 bloods should preferably be taken within 7 days prior to randomisation/C1 date, but bloods taken up to a maximum of 9 days pre-randomisation/C1 date will be accepted
(8) From cycle 2 onwards, pre-cycle bloods should ideally be performed within 3 days of day 1 of a treatment cycle, however bloods performed within a maximum of 5 days of day 1 of a treatment cycle will be accepted. Bloods taken for a follow-up visit may be completed within one month prior to the scheduled visit. Pre-cycle (excluding pre-randomisation) blood tests may be completed locally / at a patient's GP. For years 6-9 follow-up visits, only CEA is required. The blood test results must be signed dated and filed in patient notes for source verification.
(9) At least one of AST or ALT should be performed to assess liver function.
(10) The maximum CEA level accepted for randomisation is 1.2 times the upper limit of normal, relating to a site specific normal range.

(11) CEA should be performed 6-weekly during treatment. For patients allocated to 12 wks XELOX this should at cycles 2 and 4, and for patients allocated to 24 weeks XELOX this should be at cycles 2, 4, 6 and 8.
(12) CEA should be performed 6-weekly during treatment. For patients allocated to 12 wks OxMdG this should be at cycles 3 and 6, and for patients allocated to 24 weeks OxMdG this should be at cycles 3, 6, 9 and 12.
(13) CT scan is the preferred method of radiological assessment, however it is acceptable to use US of liver and CXR combined as a substitute except at baseline, mth 12 and mth 24. The baseline CT scan should be performed within 16 weeks of the date of randomisation, however CT scans dated to a maximum 16 weeks and 5 days pre-randomisation date will be accepted.
(14) CT Scans performed up to 42 days prior to the scheduled follow up visit date will be accepted and used for that follow up visit.
(15) Only patients who were randomised to 12 weeks of treatment should complete the questionnaires at the Month 6 follow up visit.
(16) **EORTC QLQ-C30 & CR29 and EQ-5D questionnaires** - Only applies to patients randomised prior to 16Feb2011: Participation in patient questionnaires was an optional part of the study (centres opted in or out of this at site randomisation). It is acceptable for baseline questionnaires to be completed on day 1 of cycle 1. Any patients randomised prior to this date must continue to complete these questionnaires at the required time-points as per the SCOT protocol**.**
(17) **GOG NTX 4 questionnaire** – Applies to patients randomized prior to 16Feb2011 and **post approval of version 4.0 of the protocol**. Participation in patient questionnaires was an optional part of the study (centres opted in or out of this at site randomisation). It is acceptable for baseline questionnaires to be completed on day 1 of cycle 1. Any patients randomised prior to this date must continue to complete these questionnaires at the required time-points as per the SCOT protocol**.**
(18) Post original to OCTO/CTU Glasgow within 1 month of completion
(19) Only required for those patients identified at randomisation as having high risk stage II disease (T3 N0 or T4 N0).
(20) For those patients who have consented to tumour sample collection, paraffin embedded tumour tissue obtained at surgical resection of the primary tumour prior to entry to SCOT should be collected and sent to the CRUK CTU Glasgow. Collection packs will be provided and further instructions will be available in a separate transSCOT translational research manual.
(21) For those patients who have consented to blood sample collection. Bloods should be collected prior to the start of therapy, however for existing patients; bloods can be collected at any point during treatment or Follow Up and sent to the OCTO Trials Office. Collection packs will be provided and further instructions will be available in a separate transSCOT translational research manual.
(22) For FU visits year 4 and onwards patients may be followed up remotely. Blood tests may be completed locally / at the local GP - as per footnote 8. The FU CRF may be completed by a phone call to the patient and review of the patient notes.

CONTENTS

1 INTRODUCTION 14
1.1 BACKGROUND 14
1.2 RATIONALE 17
2 STUDY OBJECTIVES 18
3 STUDY DESIGN 18
3.1 TYPE OF STUDY 18
3.2 ENROLMENT 18
3.3 PATIENT RANDOMISATION (NO LONGER RECRUITING PATIENTS) 18
3.3.1 Upfront Randomisation Method 18
3.3.2 Delayed Randomisation (No longer in use on the SCOT Study) 19
3.4 DURATION OF STUDY 21
4 PATIENT SELECTION 21
4.1 RANDOMISATION EVALUATIONS (BASELINE) 21
4.2 INCLUSION CRITERIA (BASELINE) 22
4.3 EXCLUSION CRITERIA (BASELINE) 23
4.4 STOPPING ALLOCATED SCOT TREATMENT EARLY 23
4.5 CONSENT WITHDRAWAL 23
5 TREATMENTS 24
5.1 TREATMENT REGIMENS 24
5.1.1 Dose and Administration of XELOX – 3 weekly cycle 24
5.1.2 Dose and Administration of OxMdG - 2 weekly cycle 25
5.2 DOSE MODIFICATIONS FOR TOXICITY 25
5.3 PRE-MEDICATION 29
5.4 INTERACTION WITH OTHER DRUGS 29
5.5 USE OF CALCIUM AND MAGNESIUM SUPPLEMENTS 29
6 MANAGEMENT AND HANDLING OF INVESTIGATIONAL MEDICINAL PRODUCT (IMP) 30
7 SAFETY ASSESSMENT 30
7.1 DEFINITION OF AN ADVERSE EVENT 30
7.2 ADVERSE EVENT REPORTING 30
7.3 DEFINITION OF AN ADVERSE REACTION 31
7.4 ADVERSE REACTION RECORDING 31
7.5 DEFINITION OF A SERIOUS ADVERSE EVENT 31
7.6 DEFINITION OF A SERIOUS ADVERSE REACTION 32
7.7 DEFINITION OF SUSPECTED UNEXPECTED SERIOUS ADVERSE REACTIONS (SUSARS) 32
7.8 DEFINITION OF NIMPS (NON INVESTIGATIONAL MEDICINAL PRODUCTS) 32
7.9 WHEN SAES ARE NOT REQUIRED TO BE REPORTED 33
7.10 PROCEDURE FOR REPORTING SAES 33
7.11 PROCEDURE FOR IDENTIFYING SUSARS 34
7.12 PROCEDURE FOR REPORTING SUSARS 34
7.14 ANNUAL SAFETY REPORTS. DEVELOPMENT SAFETY UPDATE REPORTS 34

7.15 Reference Safety Information 35
8 ASSESSMENT AND FOLLOW-UP 35
8.1 Quality of Life Questionnaires 37
8.2 Pregnancy 38
9 STATISTICS AND DATA ANALYSIS 38
9.1 Sample Size 38
9.2 Analytical Plan 39
9.2.1 Primary efficacy analysis 39
9.2.2 Secondary efficacy analysis 39
9.2.3 Safety Analysis 39
9.2.4 Quality of life analysis 39
9.2.5 Comparison of randomisation time points 39
9.2.6 Interim Analysis 40
9.2.7 Economic Analyses 40
10 TRANSLATIONAL RESEARCH 41
11 REGULATORY ISSUES 42
11.1 Clinical Trial Authorisation (CTA) 42
11.2 Ethics Approval 42
11.3 Informed Consent 42
11.4 Confidentiality 43
11.5 Liability, Indemnity and Insurance 43
11.6 Funding 43
11.7 Monitoring 44
11.8 Audits and Inspections 44
11.9 Recruitment to Other Research Studies 44
12 TRIAL MANAGEMENT AND DATA COLLECTION 44
12.1 Study Organisation 44
12.2 Study Start Up 45
12.2.1 Core Documents 45
12.3 Data Collection 45
12.4 Follow-up 46
12.5 Trial Management Group (TMG) 47
12.6 Trial Steering Committee (TSC) 47
12.7 Data Monitoring and Ethics Committee (DMEC) 47
13 ALLOCATION OF STUDY RESPONSIBILITIES 47
13.1 Sponsor Responsibilities (GGHB/GU) 47
13.2 CR-UK Clinical Trials Unit (CTU) and Oncology Clinical Trials Office (OCTO) 47
13.3 Chief Investigator (CI) 47
13.4 Participating Site 48
13.5 Principal Investigator (PI) 48
14 QUALITY ASSURANCE/QUALITY CONTROL 48
15 DEFINITION OF END OF STUDY 49
16 PUBLICATION POLICY AND AUTHORSHIP 49

17 REFERENCES 50
APPENDIX 1: WHO PERFORMANCE STATUS 53
APPENDIX 2: COCKCROFT-GAULT EQUATION 54
APPENDIX 3: CAPECITABINE DOSE BANDING 55
APPENDIX 4: NEW YORK HEART ASSOCIATION STAGING (NYHA) 56
APPENDIX 5: EXPECTED TOXICITY PROFILE OF STUDY MEDICATIONS 57
APPENDIX 6: EORTC QLQ-C30 & CR29 (COLORECTAL MODULE) 59
APPENDIX 7: GOG NTX 4 QUESTIONNAIRE 63
APPENDIX 8: EQ-5D QUESTIONNAIRE 64
APPENDIX 9: DECLARATION OF HELSINKI 66
APPENDIX 10: PATIENT WITHDRAWAL QUESTIONNAIRE 69
APPENDIX 11: SCOT TUMOUR STAGING GUIDELINE 70

1 INTRODUCTION

1.1 Background

Colorectal cancer is the second leading cause of cancer mortality in the UK. In 2001 there were approximately 34,500 new cases and 16,200 deaths from colorectal cancer[1]. Following a complete surgical resection, patients face a 40-50% chance of disease relapse[2]. With the exception of a small proportion of patients who will have disease amenable to further curative surgical resection, recurrence will lead to death.

Over the last three decades many large prospective, randomised clinical trials have established a benefit for adjuvant 5-fluorouracil (5-FU) - based chemotherapy for stage III disease. A reduction in mortality in the order of 30-35% (relative risk reduction) in favour of chemotherapy versus surgery alone has been shown in several studies. This translates into an approximately 10% absolute improvement in survival at 5 years[3-9]. Adjuvant chemotherapy is now accepted as standard practice in this group of patients providing there are no contraindications to therapy.

The benefit for patients with node negative (Stage II) disease is less clear. Two recent meta-analyses found no direct evidence of a statistically significant benefit for chemotherapy in Stage II patients[10, 11]. However, the QUASAR study recently reported a statistically significant improvement in the risk of recurrence (22.2% v 26.2%) and survival (80.3% v 77.4%) with adjuvant therapy for patients with stage II disease[12]. The MOSAIC investigators reported a post hoc subgroup analysis of patients with Stage II disease. The 3 year disease free survival rates were 87.0% with FOLFOX4 and 84.3% with 5-FU/LV, with a hazard ratio of 0.80 (95%CI, 0.56-1.15)[13]. Although this was not statistically significant, the improvement in 3 year DFS is more evident in the subgroup of patients deemed to have high risk disease; 84.9% with FOLFOX and 79.8% with 5-FU-LV (HR 0.72, CI 0.48-1.08). High risk is defined as T4 disease, perforation, obstruction, <10 nodes examined, poorly differentiated histology or extramural vascular invasion.

This recent evidence suggests that there may be a small survival benefit for adjuvant therapy in Stage II disease. On the basis of the MOSAIC data this may translate into an absolute gain of about 8% with oxaliplatin based regimens when compared to no treatment, particularly in those patients with high risk features. It is current practice in the UK to discuss the option of chemotherapy with such patients, whilst outlining the potential toxicities.

One of the drawbacks of 5-FU chemotherapy is the need for repeated intravenous infusion. The oral 5-FU pro-drug capecitabine has recently been studied in the adjuvant setting. The X-ACT study randomly assigned patients with resected stage III disease to receive capecitabine (1250mg/m^2 twice daily for 14 days, every 3 weeks), or iv 5-FU/LV Mayo clinic regimen (LV 20 mg/m^2 + 5-FU 425 mg/m^2 days 1-5, every 4 weeks x 24 weeks). The study was designed to show non-inferiority of capecitabine but in fact showed a trend towards superiority of capecitabine in both disease free survival (HR 0.87, CI 0.75 – 1.0) and overall survival (HR 0.84, CI 0.69 - 1.01)[14]. The safety data show that with the exception of hand foot syndrome capecitabine has a significantly better safety profile than 5-FU. This remained the case when a subgroup analysis of older patients (>65 years) was performed[15]. This study suggests that oral capecitabine can be substituted for intravenous 5-FU monotherapy.

With strong evidence for the efficacy of the newer chemotherapy agents (oxaliplatin and irinotecan) in the metastatic setting, several studies have included these agents in the adjuvant setting. The aforementioned MOSAIC trial showed a significant increase in 3 year disease free survival in the oxaliplatin/LV5FU2 combination arm (78.2% v 72.9%, all stages of disease,) compared with LV5FU2 alone[13]. This benefit is at expense of increased toxicity. Neutropenia, diarrhoea, vomiting and peripheral neuropathy were more common in the oxaliplatin/LV5FU2 arm, but the rate of febrile neutropenia was only 1.8% and rate of death

during treatment was similar in both groups at 0.5%. Grade 3 peripheral neuropathy occurred in 12.4 % of patients receiving oxaliplatin, but by one year post treatment this had fallen to 1.1%.

The NSABP C-07 trial used the FLOX regimen, oxaliplatin in combination with 5-FU/LV (500mg/m^2 i.v. bolus weekly x 6, each 8 week cycle x 3) versus 5-FU/LV alone. The 3 year disease free survival was 76.5% and 71.6% respectively. This gives a hazard ratio of 0.79 (95% CI 0.67 - 0.93) in favour of FLOX. [(16)]

The strongly positive nature of these studies has led to oxaliplatin/5FU being adopted as the standard of care in many countries for Stage III disease. Given the evidence with capecitabine as monotherapy in the adjuvant setting and the reported studies of the combined use of oxaliplatin and capecitabine in the metastatic setting, the next logical step is to study oxaliplatin/capecitabine as adjuvant treatment. A number of studies are underway which include this combination.

Three large studies using irinotecan in combination with 5-FU have failed to show a benefit for irinotecan in the adjuvant setting. The CALGB 89803 trial randomly assigned stage III patients to receive irinotecan, 5-FU and LV (IFL) or a bolus 5-FU/LV regimen. IFL showed no improvement in overall survival (HR 0.88) or failure free survival (HR 0.81). Toxic death was greater in the IFL group at 2.7 %[(17)]. The recently reported PETACC 3 trial failed to show a statistically significant difference between irinotecan/LV5FU2 and LV5FU2 alone with regard to 3 year disease free or relapse free survival (HR 0.89 and 0.87 respectively)[(18)]. This study did not show the increased toxic death rate seen in CALGB 89803.

The preliminary results from the Accord 02 study showed no improvement in event free survival in the irinotecan/LV5FU arm compared with the LV5FU alone arm. However the two arms were unbalanced with more T3/4, more heavily node positive (>15 nodes) and more vascular invasion in the irinotecan-containing arm[(19)]. In light of these surprising findings a number of current and proposed trials using irinotecan have been halted. This has left a gap in the adjuvant trial repertoire.

Novel approaches to the treatment of colorectal cancer are being explored. Two biological agents, bevacizumab, a monoclonal antibody targeting vascular endothelial growth factor (VEGF), and cetuximab, a monoclonal antibody which specifically blocks the epidermal growth factor receptor (EGFR) have been studied in the metastatic setting. A study of irinotecan/5-FU plus or minus bevacizumab as first line treatment in metastatic colorectal cancer reported an increased median duration of survival from 15.6 months with IFL to 20.3 months with IFL plus bevacizumab (HR for death 0.66)[(20)]. A specific toxicity profile is associated with bevacizumab, which is distinct from chemotherapy, and includes hypertension, bleeding diathesis, proteinuria and gastrointestinal perforation.

Published data on the use of cetuximab monotherapy or in combination with irinotecan in patients with irinotecan-refractory disease have shown an overall response rate of 22.9% in the combination arm and 10.8% in the monotherapy arm. The median time to progression was 4.1 months in the combination arm versus 1.5 months for the monotherapy arm (HR 0.54, CI 0.42 – 0.71 for progression)[(21)]. The main additional toxicity of cetuximab is an acneform skin rash, the severity of which relates to response.

An observed tumour response rate of 22.9% in patients who have failed on up to three standard treatments in the metastatic setting is clinically important and has led to the incorporation of biological agents into adjuvant treatment protocols to ascertain whether this benefit can be translated to the adjuvant setting. The NSABP-C08 and the AVANT studies have not shown any benefit when bevacizumab was added to adjuvant FOLFOX / CAPEOX. However the ongoing QUASAR 2, study is investigating whether adding this agent to single agent capecitabine does bring about some benefit.

The advent of multi-agent adjuvant clinical trials has raised a number of issues which require to be addressed by the oncology community worldwide. The addition of oxaliplatin and biological agents has led to increased toxicity of treatment as already described. This is an important issue when dealing with a population of patients in whom a proportion will be cured of their disease by surgical intervention alone. The particular concern with oxaliplatin is the potential for long term neurotoxicity and associated functional impairment. The original Phase I study of oxaliplatin showed that there was a clear relationship between cumulative dose and incidence and severity of neuropathy[22]. This has been borne out in clinical practice. The Intergroup trial N9741 reported a detailed analysis of oxaliplatin-associated neurotoxicity in metastatic patients. This analysis found that 62% of patients withdrew from treatment with FOLFOX for reasons other than disease progression. Of the patients who were available for analysis 23% dropped out due to myelosuppression and 23% due to neurotoxicity. The time to grade 2 or 3 neurotoxicity was clearly related to duration of treatment. Interestingly 92% of patients who responded to treatment had done so at a cumulative dose of 680mg of oxaliplatin (7.5 cycles of treatment), and 96% had done so at 800mg (9.5 cycles). The incidence of neuropathy was maximal at the maximal dose of oxaliplatin (1020mg, 12 cycles)[23]. This suggests that perhaps the benefit of treatment for the vast majority of patients could be achieved with a shorter treatment schedule therefore reducing the incidence of debilitating neuropathy.

Another important issue, which thus far has been little investigated, is that of the escalating cost of treatment and the cost effectiveness of such treatments. The estimated cost of eight weeks of treatment for metastatic disease with the Mayo Clinic regime of 5-FU is US$ 63, with FOLFOX is US$ 11,889 and for FOLFOX plus bevacizumab is US$ 21,033[24]. These costs are for drug only and do not include the cost of administration, supportive medications or treating complications. The MOSAIC and X-ACT trials have both reported data relating to resource saving and cost effectiveness of treatment. The MOSAIC trial extrapolated within-trial data to estimate a benefit in overall life expectancy. This was estimated to be 1.17 years benefit in the oxaliplatin/LV5FU2 arm compared with LV5FU2 alone. The cost per life year gained was estimated to be US$ 27,300[25]. The X-ACT study reported medical resource utilisation data which showed substantial savings due to the avoidance of i.v. therapy administration costs with oral capecitabine[26].

As newer and more expensive drugs are incorporated into the adjuvant treatment armamentarium, the costs of such treatment will exert enormous pressure on healthcare resources. Analysis of the cost-effectiveness of treatments will become increasingly important and strategies to maximise cost-effectiveness will have to be investigated.

One way to address these important issues of increasing toxicity and cost would be to shorten the duration of treatment. The evidence base for reducing the duration of treatment is small but the rationale for testing the hypothesis can be supported by inference from a number of sources. Historically 5-FU/FA was given as adjuvant treatment for 12 months. Subsequent investigation demonstrated no statistically significant reduction in efficacy of 6 months treatment compared to 12 months, and 6 months treatment became the standard of care[5]. The SAFFA trial compared six months of standard Mayo clinic bolus 5-FU/FA with 3 months of protracted venous infusion of 5-FU (PVI-5FU). This study was somewhat underpowered, but reducing the treatment duration was not associated with any observed detrimental effect on outcome. Furthermore, the short course treatment was associated with less toxicity and better quality of life than standard bolus treatment[27]. Capecitabine provides prolonged 5-FU exposure at lower peak concentration than bolus regimens and simulates PVI-5FU. Given the favourable equivalence and toxicity data from the X-ACT trial it would be a reasonable hypothesis that capecitabine could be substituted for PVI-5FU without compromising outcome.

As already described, the Intergroup trial N9741 reported that 92% of patients with metastatic disease who had responded, had done so having had just over half of the planned chemotherapy cycles[23]. This may suggest that shorter duration of treatment is feasible but as yet has not been directly studied.

This study also aimed to answer an important question in clinical trial methodology, in terms of the randomisation time-point in studies where the aim is to reduce standard treatment duration. All patients entering this study will be randomised to receive either 12 weeks or 24 weeks of treatment.

To address this question of randomisation timing, during the first year of study recruitment, centres were randomised to use one of the two randomisation time-points. Randomisation was performed at baseline for patients being randomised upfront, or after 12 weeks of treatment for patients on the delayed randomisation time-point (patients who are fit to continue were randomised either to stop or continue treatment for a further 12 weeks). All patients on the delayed randomisation time-point were registered and their eligibility confirmed at baseline.

The latter randomisation approach is statistically the optimal approach, especially in this non-inferiority setting, as it excludes from the treatment comparison in an unbiased way patients who would never receive treatment beyond 12 weeks (for toxicity, early progression etc). Such patients add nothing to the question of comparing 12 weeks of treatment with 24 weeks of treatment and simply reduce the power of the study to detect differences.

This approach has been attempted previously in a non-inferiority study seeking to reduce the length of the treatment in non-small cell lung cancer [28] and was abandoned when only 4 of the first 17 patients opted for randomisation, the remainder wishing to continue therapy.

Whilst randomisation at 12 weeks is the statistically more robust option, it was thought that this would be more difficult to implement in clinical practice. The uncertainty about the length of treatment may be less appealing to the patient and interfere with their ability to plan their life post treatment. This may have a significant impact on the study's ability to recruit the necessary number of patients in a reasonable time frame.

The Short Course Oncology Therapy (SCOT) trial aims to address a number of the issues outlined above. Firstly, is 12 weeks of adjuvant chemotherapy for colorectal cancer equivalent in terms of efficacy to 24 weeks of treatment? Secondly, are the immediate and long term toxicity and quality of life profiles improved by a shorter course of treatment? Thirdly, what are the implications in terms of cost, both in terms of drug costs and resource utilisation? Finally is it possible to randomise at 12 weeks and still achieve adequate recruitment and compliance to achieve the study objectives.

At the end of the first year of recruitment the independent members of the Trial Steering Committee (TSC) reviewed the data presented in the first interim analysis report. It was noted that although the rate of recruitment was relatively constant between the two time-points, it was found that there was a high drop out rate of 32% of patients prior to the delayed week 12 randomisation, compared to a 7% dropout of patients stopping treatment before completing 12 weeks of treatment on the upfront randomisation arm. The TSC felt this dropout rate to be the single most important parameter in making a decision regarding the two randomisation time points. As a result, the decision made by the independent members of the TSC was that the study should continue with the upfront randomisation time point only, for the duration of the study. This was implemented across all study sites on 6th July 2009. From this date, all patients entered on to the study are to be randomised upfront to either 12 or 24 weeks of treatment.

1.2 Rationale

Currently the standard treatment duration for adjuvant chemotherapy for colorectal cancer is 24 weeks.

The research questions which this study has been designed to answer are –

- Is 12 weeks of adjuvant treatment for colorectal cancer equivalent to 24 weeks in terms of disease-free and overall survival?
- Is 12 weeks of treatment less toxic than 24 weeks?
- Are there cost savings from reducing treatment duration and which treatment option represents the best value for money?
- Is it possible to randomise at 12 weeks and still achieve adequate recruitment and compliance to achieve study objectives? (Please see section 3 for further information)

2 STUDY OBJECTIVES

There are 3 main study objectives:-

- Assessment of the efficacy of 12 weeks of treatment versus 24 weeks of treatment and comparison of the associated toxicity.
- Economic analysis to assess the cost effectiveness of the two treatment alternatives.
- Comparison of two randomisation methodologies (Please see section 3.3 for further information).

- **Primary endpoint:**
  Disease–free survival (defined as time from randomisation to recurrence, development of new colorectal cancer or death from any cause).

- **Secondary endpoints:**
  Overall survival, cost-effectiveness, toxicity, and quality of life.

3 STUDY DESIGN

3.1 Type of Study

This is a Phase III, open-label, randomised controlled, two arm, multi-centre, non-inferiority trial.

3.2 Enrolment

Recruitment ended on the 29th November 2013. The trial randomised 6087 patients with fully resected stage III or fully resected high-risk stage II colorectal cancer to receive either 24 or 12 weeks of adjuvant chemotherapy over 5.5 years. The treatment regimen was either oxaliplatin/5FU (OxMdG) or oxaliplatin/capecitabine (XELOX). Each individual participating site was able to select which treatment regimen they wished to use for each individual patient prior to randomisation.

3.3 Patient Randomisation (No longer recruiting patients)

3.3.1 Upfront Randomisation Method

Patients will not be able to be randomised to the study until the site has been activated.

From the 06th of July 2009 onwards, all patients are randomised to the SCOT study using the upfront randomisation method. At upfront randomisation, patients are allocated to either 24 or 12 weeks of treatment prior to commencing adjuvant chemotherapy.

Randomisation must occur prior to a patient commencing treatment. A patient's eligibility must be confirmed and the consent and registration/randomisation forms must be completed prior to site staff contacting the relevant coordinating trials centre to randomise the patient to the study. Randomisation to the study can be done by either telephone or fax.

Cancer Research UK Clinical Trials Unit, Glasgow

Randomisation Telephone Number: 0141 301 7195
Randomisation Fax Number: 0141 301 7192

Randomisation Service (UK opening hours):
Monday- Thursday 08.30-17.00
Friday 08.30-16.30
Fax 24 hours*

From Quarter 2-3 of 2012 (21st August 2012) all participating sites in the SCOT trial must contact their coordinating trials office (OCTO or CR-UK CTU Glasgow as appropriate) directly to randomise patients to the SCOT trial. CR-UK CTU Glasgow randomisation contact details will remain as per the above and those sites coordinated by OCTO should contact OCTO directly to randomise on the following numbers

Oncology Clinical Trials Office (OCTO), Oxford

Randomisation UK Telephone Number 0800 389 1635

Randomisation UK Fax Number 0800 389 1629

Randomisation Non UK Telephone Number: +44(0)1865 617 014
Randomisation Non-UK Fax Number: +44(0)1865 617 015

Randomisation Service (UK opening hours):
Monday - Friday – 09:00 – 17:00
Fax 24 hours*

* Faxes received outside office hours will be dealt with the next working day

All patients must be randomised onto the study prior to commencement of any treatment.

Each patient randomised will be allocated a unique study identifier and will be given the treatment duration allocation (24 or 12 weeks) at this point.

A minimisation algorithm incorporating a random component will be used to allocate patients to treatment durations; the factors used in the minimisation will be centre, choice of regimen, gender, disease site, N-stage and T-stage and if patient is to start the XELOX regimen the starting dose of capecitabine will also be a stratification factor.

3.3.2 Delayed Randomisation (No longer in use on the SCOT Study)

Prior to the 06th July 2009 site Principal Investigators (PIs) participating in the study were randomly allocated to one of two randomisation time points.

1) Upfront Randomisation - at commencement of adjuvant treatment to either 24 or 12 weeks of chemotherapy.
2) Delayed Randomisation - after 12 weeks of adjuvant treatment to either stopping treatment at that point, or continuing with a further 12 weeks of chemotherapy.

By randomising the PIs, this ensured that PIs, who had more than one site, were allocated the same randomisation time point across the sites. This allocation was stratified for estimated total recruitment across all the listed PI sites.

Each PI was randomised to one of the above randomisation time points via completion of the PI Randomisation Method Questionnaire. This questionnaire was sent to the PI (or designee) for completion when at least local ethical approval, R&D approval and the Clinical Trial Agreement were in place. For PIs responsible for more than one site, this was completed when the first of their sites met the above criteria. The questionnaire was returned by fax (0141 301 7192), or scanned and emailed (scot@clinmed.gla.ac.uk) to the Cancer Research UK Clinical Trials Unit, Glasgow (CTU). PIs were then informed of their randomisation time point as soon as possible after this was submitted and before site activation.

Sites that were allocated to randomise patients after 12 weeks of treatment (delayed randomisation) registered their patients, prior to commencement of treatment, as per the procedure detailed in section 3.3.1 and went on to be randomised after 12 weeks of treatment to receive no further treatment or 12 weeks further treatment.

Sites randomising patients at week 12 had to complete a week 12 randomisation form. It was preferred that patients were randomised as close as possible to the week 12 time point but it was accepted that for some sites this may have caused logistical problems. In these cases, randomisation was accepted prior to week 12 but this could only take place <u>after</u> administration of the last scheduled cycle of treatment (post cycle 4 of XELOX or cycle 6 of OxMdG).

<u>**Sites were requested to note the following**</u>:

- If either of the two treatment durations was not acceptable to the <u>patient or clinician, the week 12 randomisation was not to be performed.</u> These patients would not be randomised at week 12 and received no further SCOT treatment or SCOT follow-up.

- If patients had previous dose modifications, patients could still be randomised at week 12 as long as the clinician was happy for the patient to potentially receive either of the treatment durations.

- Any other queries with respect to week 12 randomisation that did not fall into these categories above were checked on a per patient basis with the coordinating centres.

If the decision was made not to randomise a patient at week 12, the week 12 randomisation form was still completed to confirm the reason why the randomisation was not performed.

A minimisation algorithm incorporating a random component was used to allocate patients to treatment durations; the factors used in the minimisation were centre, choice of regimen, gender, disease site, N-stage and T-stage.

From the outset of the study it was specified that a decision would be made in relation to the two randomisation time points approximately 1 year after the study opened to recruitment. At the Trial Steering Committee (TSC) meeting held on the 16th June 2009, the independent members of the TSC came to a decision on this based on the data presented in the first interim analysis report. In particular, in relation to the two randomisation time points, it was noted that there was a high drop out of 32% of patients prior to the delayed week 12 randomisation, compared to a 7% dropout of patients stopping treatment before completing 12 weeks of treatment on the upfront randomisation arm. The TSC felt this dropout rate to be

the single most important parameter in making a decision regarding the two randomisation time points. As a result, the decision made by the independent members of the TSC was that the study should continue with the upfront randomisation time point only, from the 06th July 2009.
After the TSC decision, all patients registered/randomised to the trial using the delayed randomisation method were provided with a letter explaining that only the upfront randomisation PIS would be updated moving forward (including version 4.0 onwards) and if appropriate they will receive copies of the upfront randomisation PIS if/when updates are necessary for the duration of the trial.

3.4 Duration of study

The trial was due to close in February 2015, with all patients having a least one year of follow-up (with follow-up ending at the end of November 2014). Following a successful application to the funder (outcome November 2015), all Stage III patients should now be followed up for a minimum of 3 years (for the last stage III patient recruited this means the last follow-up is 30 November 2016) and all Stage II patients should be followed up to the end of November 2017. Prior to the approval for the extension of follow up from the funder, there was an interim request to sites to follow up all Stage II patients as per protocol and for Stage III patients yet to reach the 3 year follow up time point until at least November 2015. The reason for this extension is to observe more relapses and thus improve study power, as the study fell below its original recruitment target.

Registration of patients with Medical Research Information Services (MRIS) at the NHS Information Centre for Health and Social Care will enable survival follow-up beyond the specified 9 year period.

A one off update on patient status will be collected on 30th November 2018. Sites will be asked to complete a form to give the following:

- Survival data on all patients previously known to be alive
- For patients who have no record of relapse, any details of subsequent relapse

4 PATIENT SELECTION

4.1 Randomisation Evaluations (Baseline)

Prior to randomisation at baseline, the patient must have fully recovered from surgery and be deemed suitable for adjuvant chemotherapy. The authorised pathology report must confirm microscopically clear surgical resection margins (R0 which is defined as >1mm clearance). Staging assessments should be performed within 16 weeks prior to randomisation (however staging assessments up to a maximum of 16 weeks and 5 days will be accepted) and will consist of a CT scan of the chest, abdomen and pelvis.

A complete colonoscopy should be performed either pre-surgery or post-surgery. Pre-surgery colonoscopies up to a maximum of 6 months prior to surgery are acceptable. Post-surgery colonoscopies should preferably be performed within 3 months, but at the very least within 12 months to rule out synchronous primaries. If a complete colonoscopy is not performed then another investigation to completely visualise the entire colon must be performed (e.g. virtual CT colonoscopy). A flexible sigmoidoscopy will be considered as acceptable if a previous CT scan has indicated that this would be a sufficient method of investigation.

If a patient has two (or more) synchronous tumours then the histopathological staging of the most severe tumour will be used to determine eligibility, i.e. as long as that tumour fulfils the staging criteria (and in the absence of metastatic disease) then the patient is still potentially eligible for the study as long as all other eligibility criteria are fulfilled.

CEA should be normal (as per local values) at the time of randomisation. If the CEA level is ≤20% above the local ULN (1.2 X ULN), the patient will still be considered to be eligible providing that there is no evidence of residual or metastatic disease and all other eligibility criteria are met.

Assessments made up to 7 days before randomisation include:
(details of assessments (and any waivers if applicable) must be documented in the hospital notes)

- Medical history and Physical/Clinical examination, including height and weight.
- Body Surface Area
- WHO performance status.
- ECG*
- Baseline laboratory tests** including CEA, full blood count, liver function tests (AST or ALT), renal function tests (including creatinine clearance calculation). INR and APTT must be performed for patients on concurrent treatment with anti-coagulants.
- Urea & Electrolytes**
- Urine pregnancy test for all women of childbearing potential**

* A baseline ECG should ideally be performed within 7 days prior to randomisation, however ECGs performed up to 20 days prior to randomisation will be accepted.

**Baseline blood/urine pregnancy test results should ideally be obtained within 7 days prior to randomisation; however blood/urine pregnancy tests taken up to 9 days prior to randomisation will be accepted.

Chemotherapy treatment must commence within a maximum of 13 weeks post-surgery (see below for details).

Once the patient has been randomised to the trial, if the patient has consented to their GP being notified, the current version of the appropriate GP letter (enclosing information regarding potential side effects and problematic medications) must be sent to the patient's GP.

4.2 Inclusion Criteria (Baseline)

- Fully resected stage III colorectal cancer*

  Or,

  Fully resected high-risk stage II colorectal cancer* (defined as having one or more of the following - T4 disease, tumour obstruction and/or perforation of the primary tumour during the pre-operative period, inadequate nodal harvest as indicated by <10 nodes examined, poorly differentiated histology, perineural invasion, peritoneal involvement or extramural venous/lymphatic invasion).
  See tumour staging guideline in Appendix 11 for clarification on SCOT eligibility.
- No evidence of residual or metastatic disease.
- Ideally patients should be randomised within 11 weeks of surgery and treatment should start within 2 weeks of randomisation. However as long as the surgery to cycle 1 treatment start date is ≤ 13 weeks the patient will be considered eligible.
- WHO PS = 0 or 1.
- Age ≥18 years.
- Life expectancy >5 years with reference to non-cancer related diseases.
- Written informed consent.
- CEA ≤ 1.2 X ULN (as per local values) (see Schedule of Assessments section 4.1 Baseline Evaluations for details).

- Patients with rectal cancer will be eligible unless they have had pre-operative combined chemotherapy and radiotherapy, or are scheduled for post-operative combined chemotherapy and radiotherapy.
- *All rectal cancer patients* included in the trial must have had TME type surgery with negative (R0) resection margins (R0 defined as greater than 1mm clearance).

* All patients must have negative (R0) resection margins defined as greater than 1mm clearance.

4.3 Exclusion Criteria (Baseline)

- Previous chemotherapy*.
- Previous long course chemoradiotherapy (pre-operative short course radiotherapy is allowed).
- Moderate/severe renal impairment (GFR/Creatinine Clearance <30 ml/min), as calculated by the Cockcroft-Gault equation (Appendix 2).
- Absolute neutrophil count<$1.5x10^9$/L.
- Platelet count <$100x10^9$/L.
- Haemoglobin <9 g/dL.
- Aspartate aminotransferase/Alanine aminotransferase >2.5 x upper limit of normal (at least one of AST or ALT must be performed).
- Clinically significant cardiovascular disease. [i.e. active; or <12 months since e.g. cerebrovascular accident, myocardial infarction, unstable angina, New York Heart Association (NYHA – Appendix 4) grade II or greater congestive heart failure, serious cardiac arrhythmia requiring medication, uncontrolled hypertension].
- Pregnancy/lactation or of child bearing potential and not using, or willing to use medically approved contraception. (Postmenopausal women must have been amenorrheic for at least 12 months to be considered of non-childbearing potential.)
- Previous malignancy other than adequately treated in situ carcinoma of the uterine cervix or basal or squamous cell carcinoma of the skin, unless there has been a disease-free interval of at least 5 years.
- Known or suspected dihydropyrimidine dehydrogenase (DPD) deficiency.

* No previous chemotherapy except chemotherapy administered with curative intent completed more than 5 years ago and from which there are no residual complications.

4.4 Stopping Allocated SCOT Treatment Early

If the patient stops their allocated treatment early, a Patient Withdrawal Questionnaire should be completed by the patient to provide specific information on the reasons for stopping their allocated SCOT treatment early.
Details of all patients who stop their allocated SCOT treatment early should be recorded on the Patient Withdrawal Log. See Appendix 10 for the Patient Withdrawal Questionnaire. This original completed questionnaire should be returned to the allocated coordinating trials unit. All patients affected should be followed-up as per the SCOT patient diary / protocol i.e. 24 week patients will have their first SCOT follow-up visit at month 6 post randomisation date and 12 week patients will have their first SCOT follow-up visit at month 4 post randomisation date (unless the patient decides to withdraw their consent from the trial).

4.5 Consent Withdrawal

The patient can decide to withdraw their consent from the study at any time. It will be important for the clinician looking after the patient to determine whether the patient **just wants to stop protocol treatment** or if they are withdrawing their consent to undertake any further study procedures including follow-up. If the patient definitely wants to withdraw consent for the study, then they should be asked to complete a 'Consent Withdrawal Form'. If

a Consent Withdrawal Form is completed by the patient then a Consent Withdrawal Notification Form should be completed by the site and sent to the trial co-ordinating office.
The clinician caring for the patient also has the right to stop protocol treatment for individual patients if he/she feels that it is in the best interests of the patient. However this is not the same as withdrawing consent for the study and the patient should still undergo the remaining study procedures and follow-up if these seem appropriate. If the patient has withdrawn their consent from the study, a Patient Withdrawal Questionnaire should be completed by the patient to ask for specific information on the reasons for consent withdrawal. Details of all patients who decide to withdraw their consent should be recorded on the Patient Withdrawal Log. See Appendix 10 for the Patient Withdrawal Questionnaire. This original completed questionnaire should be returned to the allocated coordinating trials unit.
Please contact your coordinating trial office for further advice if required.

5 TREATMENTS

5.1 Treatment Regimens

Patients are randomised to either the 24 or 12 week treatment arm. The participating site will be able to select either oxaliplatin/5-FU (OxMdG) or oxaliplatin/capecitabine (XELOX) for each individual patient prior to entering the study. The above chemotherapy treatments are not supplied for the trial and sites should use own shelf/commercial stock for the trial. There are no trial specific guidelines regarding labelling for the chemotherapy treatments as the treatments are considered to be used within the product licences (detailed in Clinical Trial Authorisation (MHRA)).

- 24 weeks of XELOX/OxMdG chemotherapy
- 12 weeks of XELOX/OxMdG chemotherapy

Body surface area will be capped at $2.2m^2$ for this study. A guideline for capecitabine dose banding is provided in Appendix 3. Dose banding of oxaliplatin and IV 5-FU are permitted as per usual local practice.

5.1.1 Dose and Administration of XELOX – 3 weekly cycle

Oxaliplatin $130mg/m^2$ IV on day 1
Capecitabine $1000mg/m^2$ PO twice daily for 14 days

Oxaliplatin should be given in 250-500 ml of 5% glucose over 2 hours. Please refer to current Summary of Product Characteristics (SmPC) for instructions regarding the preparation, stability and final concentration of oxaliplatin infusion. Dose banding is permitted.

Capecitabine will be dispensed and labelled according to local practice. Dose banding is as per Appendix 3.

Patients should be advised to take the capecitabine tablets twice daily, morning and evening, with water within 30 minutes after a meal.

Patients with a creatinine clearance of 30-50mls/min must commence treatment with capecitabine at 75% of the full dose.

Patients ≥ 70 years of age should be considered for treatment with capecitabine at 75% of the full dose but, in light of differences in standard practice between sites, this will be left to the discretion of the Investigator depending on the fitness of the individual patient. The decision not to dose-reduce must be documented in the patient notes.

At the Investigator's discretion, patients can be commenced on a minimum starting dose of capecitabine of 800 mg/m^2 PO twice daily if clinically indicated. The starting dose of capecitabine will be requested at baseline randomisation.

5.1.2 Dose and Administration of OxMdG - 2 weekly cycle

Oxaliplatin 85mg/m^2 IV on day 1 concurrently with
L-folinic acid 175mg or folinic acid 350 mg followed by
5-fluorouracil 400mg/m^2 IV bolus injection over 5 minutes followed by
5-fluorouracil 2400mg/m^2 IV continuous infusion over 46 hours.

Oxaliplatin should be given in 250-500 ml of 5% glucose over 2 hours. Please refer to current SmPC for instructions regarding the preparation, stability and final concentration of oxaliplatin infusion. Dose banding is permitted.

5-FU and folinic acid should be reconstituted according to local protocols. Dose banding of 5-FU is permitted. Any brand and salt form of folinic acid may be used. However if a site chooses to switch brands during the study, the Sponsor must be informed and sites are requested to contact their coordinating trial office.

At the Investigator's discretion, OxMdG patients ≥ 70 years of age can be commenced on 5-fluorouracil IV continuous infusion and 5-fluorouracil IV bolus at 75% of the starting dose, if clinically indicated.

5.2 Dose Modifications for Toxicity

Expected toxicities are detailed in Appendix 5. Dose modifications for diarrhoea, haematological toxicity and neurotoxicity are as described below, after some general rules and observations about managing toxicity,

If any grade 1 toxicity occurs as a result of chemotherapy, then treatment will be continued, without interruption, at full dose. For all treatment-related toxicities ≥ grade 3, treatment should be withheld until recovery to ≤ grade 1 then restarted commencing as day one of the next cycle, if medically appropriate.
If patients take more than 4 weeks to recover from chemotherapy-related toxicity they will receive no further **protocol-mandated** treatment, but will still be followed up as per the SCOT protocol and their clinicians will be asked to describe what further adjuvant treatment they do receive 'off trial'. Please note a treatment delay is counted from the date the next scheduled cycle is due.

Wherever possible, oxaliplatin should be dose-reduced (as per Investigator discretion) rather than discontinued and can be given over a longer period of time if it is the hyperacute neurotoxicity which is particularly a problem. In the situation where oxaliplatin **is** discontinued due to toxicity, adjuvant treatment can continue with 5-FU or capecitabine alone if deemed appropriate by the local Investigator. In this case, the dose per m^{2}of the single agent 5-FU/capecitabine can be increased as per local practice at the discretion of the Investigator. Patients will still be considered to be on SCOT protocol treatment and will be followed-up as per the SCOT protocol.

Crossover from capecitabine to infusional 5-FU and vice versa is allowed if this is required to control toxicity. As far as is practicable, patients should be changed at a time point that keeps the trial timelines intact e.g. if a patient develops hand-foot syndrome having received 2 cycles of XELOX on the 12 week treatment arm and then the treatment is changed to OxMdG, the patient should receive a further 3 cycles of OxMdG. At all times all endeavours should be made to keep the total number of weeks of treatment as determined by randomisation. Please do not hesitate to contact your coordinating trial office for advice.

Once the dose of a specific protocol treatment has been reduced it **must not** be re-escalated.

In the event of elevated CEA levels, management should be as per local practice at the discretion of the Investigator. Please do not hesitate to contact your coordinating trial office for advice.

For toxicities or combinations of toxicities not specifically covered in detail in this protocol (see below), doses of chemotherapy can be reduced at the discretion of the Investigator as per local practice.

Any dose modifications must be recorded on the CRF and documented in the patient notes.

Haematological Toxicity

The following dose modifications are provided as a guideline in the event of haematological toxicity, however Investigators are permitted to follow their local practice for the management of haematological toxicity, with all dose modifications fully documented in the patient's medical record and CRFs.

Neutrophils		Platelets	Dose Modification
$\geq$1.3 x 10^9/l	and	$\geq$75 x10^9/l	Treat with full dose on time
<1.3 x 10^9/l	and/or	<75 x 10^9/l	Delay treatment until neutrophils and platelets are above these limits

XELOX

If > 1 delay, or 1 delay of ≥ 2 weeks occurs, reduce capecitabine and oxaliplatin doses by 25% and continue at the lower dose for subsequent cycles unless further toxicity occurs.

If further delay(s) for myelotoxicity occur despite a 25% dose reduction, further dose reductions may be made, at the discretion of the local Investigator.

OxMdG

If > 1 delay, or 1 delay of ≥ 2 weeks occurs, maintain oxaliplatin and infusional 5-FU doses , but omit bolus 5-FU and continue without bolus 5-FU for subsequent doses.

If further delay(s) for myelotoxicity occur despite omitting bolus 5-FU, reduce the oxaliplatin and infusional 5-FU doses by 25%. Further dose reductions can be made at the discretion of the local Investigator.

Following first cycle of chemotherapy

If on the day the second cycle is due neutrophils <1.0, reduce doses for subsequent courses by 25% (and omit bolus 5-FU from OxMdG regimen).

If on the day the second cycle is due neutrophils ≤0.5, Investigators may at their discretion

reduce doses for subsequent courses by 50% (and omit bolus 5-FU from OxMdG regimen). A 25% dose reduction with G-CSF would be an acceptable alternative.

XELOX and OxMdG

If delay ≥ 4 weeks patient will no longer be considered to be on SCOT protocol treatment, however the patient **will** continue to be followed up as per the SCOT protocol.

G-CSF management of neutropenia will be at the discretion of the local Investigator.

Neurosensory Toxicity

Neurosensory toxicity with these regimens is felt to be a consequence of the oxaliplatin. Therefore reduction in this drug is the most important adjustment to make. The table below gives recommendations but is not meant to be prescriptive and dose adjustments according to local protocol may be followed as long as the dose given is carefully annotated in the CRF.

Regimen	Grade 1, or 2 (if Grade 2 persisting <7 days)	Grade 2 persisting >7 days	Grade 3	Grade 4
**OxMdG**	Full dose oxaliplatin	Reduce oxaliplatin dose by 25%	Discontinue oxaliplatin	Discontinue oxaliplatin
**XELOX**	Full dose oxaliplatin	Reduce oxaliplatin dose by 25%	Discontinue oxaliplatin	Discontinue oxaliplatin

Acute dysasthesia of the larynx may be mitigated by slowing the rate of infusion of oxaliplatin.

If repeat events of neurosensory toxicity occur; local practice should be followed with the management of the toxicities being fully documented in the patient's medical records.

Diarrhoea

Diarrhoea with these regimens is a consequence of the 5FU or capecitabine. Therefore reduction in these drugs is the most important adjustment to make. The table below gives recommendations but is not meant to be prescriptive and dose adjustments according to local protocol may be followed as long as the dose given is carefully annotated in the CRF.

	Grade 2	Grade 3	Grade 4
**1st occurrence**	Withhold 5FU/capecitabine treatment until recovered to grade 0-1. Restart at full dose	Withhold 5FU/capecitabine treatment until recovered to grade 0-1. Restart with a 25% dose reduction	Consider discontinuation of all chemotherapy or if rapid recovery, treating with 50% 5FU/capecitabine
**2nd occurrence**	Withhold 5FU/capecitabine treatment until recovered to grade 0-1. Restart at 75% dose	Withhold 5FU/capecitabine treatment until recovered to grade 0-1. Restart at 50% dose	
**3rd occurrence**	Withhold 5FU/capecitabine treatment until recovered to grade 0-1. Restart at 50%	Consider discontinuation of all chemotherapy	

	dose		

If dose reduction of capecitabine or 5-FU does not result in improved tolerance then consideration should be given to reduction of oxaliplatin dose to 100 mg/m^2 on XELOX regimen or 75 mg/m^2 on OxMdG regimen.

Respiratory Toxicity

As with other platinum drugs, rare cases of acute interstitial lung disease or lung fibrosis have been reported with oxaliplatin. In the case of unexplained respiratory symptoms or signs, oxaliplatin should be discontinued until further pulmonary investigations exclude an interstitial lung disease.

Stomatitis

Routine mouthcare is recommended. If mouth ulcers occur despite this, dose reduce capecitabine or 5-FU as per table for diarrhoea.

Hand-foot Syndrome (HFS)

Treat symptomatically – Pyridoxine 50 mgs tds by mouth can be used as per Investigator discretion; or a topical corticosteroid may help. If HFS is still a problem, dose reduce capecitabine or 5-FU as per table for diarrhoea.

Allergic Reactions to Oxaliplatin

The occasional patient (approximately 0.5%) develops acute hypersensitivity to oxaliplatin, usually after more than 6 cycles have been administered. During drug administration, the patient may develop rash, fever, swollen mouth or tongue, hypo- or hypertension and other signs/symptoms of hypersensitivity. This rarely develops to full-blown anaphylaxis, even with repeated treatment.

If severe hypersensitivity occurs, discontinue the infusion and treat with IV corticosteroid and antihistamine. After full recovery, the patient may continue with folinic acid and 5-FU or capecitabine.

Specific Management of Patients taking Capecitabine

Since the commencement of the SCOT trial in March 2008, there have been nine patient deaths (correct as of 31st Jan 2011) attributed to diarrhoea, vomiting and (usually) neutropenia. These events are expected side effects of both the XELOX and OxMdG regimens.

Eight out of these nine deaths have occurred in patients receiving XELOX. Previous large scale Randomised Controlled Trials have shown that XELOX is no more toxic than other oxaliplatin/fluoropyrimidine regimens – however multiple reports exist of patients failing to discontinue the oral capecitabine in the face of toxicity, which then becomes more severe and potentially irreversible.

Patients allocated XELOX must be properly educated in the management of their home-based oral chemotherapy and need to be given rigorous advice with respect to contacting the hospital as soon as toxicities ensue.

Patients may often be prepared to experience toxicities and may not easily accept the idea of interrupting their treatment for fear this may decrease efficacy. Patients should be re-assured that protocol compliant dose modifications will not compromise the efficacy of their treatment, and must be given clear instructions on when to discontinue capecitabine and who to contact (local Investigator/Research Nurse) at the onset of key toxicities such as nausea/vomiting and diarrhoea.

5.3 Pre-Medication

Pre-medication as prophylaxis for nausea and vomiting is recommended as per local protocols. In addition, for individual patients where local vein pain is a problem during infusion, then local protocols, such as increasing infusion time, piggy-backing chemotherapy with glucose infusions etc, can be instigated.

5.4 Interaction with Other Drugs

The drugs listed below may interact with some of the IMPs given as part of this trial. We do not consider these drugs to be a recommended standard treatment for this patient group but acknowledge that there may be instances where patients receive these treatments concomitantly. If this is the case, then caution should be taken as indicated below. We do not consider these medications to be Non-Investigational Medicinal Products (NIMPs) for this trial. This list is not intended to be comprehensive, and local practice and guidelines should be followed for management of all other drugs.

Warfarin
We recommend that patients **DO NOT** receive concomitant capecitabine and warfarin as the disturbance in warfarin metabolism during capecitabine treatment is unpredictable and difficult to manage. Wherever possible we would recommend either treating the patient with low molecular weight heparin instead of warfarin, or changing the patient to OxMdG treatment rather than XELOX. If the Local Investigator feels there is no alternative to giving capecitabine and warfarin concurrently then these patients MUST have their anticoagulant response (INR or prothrombin time) monitored frequently in order to adjust the anticoagulant dose accordingly. **Altered coagulation parameters and/or bleeding, including death, have been reported in patients taking capecitabine concomitantly with coumarin-derived anticoagulants such as warfarin and phenprocoumon.**

Phenytoin
Increased phenytoin plasma concentrations have been reported during concomitant use of capecitabine with phenytoin. Formal drug interaction studies with phenytoin have not been conducted. Patients taking phenytoin concomitantly with capecitabine should be regularly monitored for increased phenytoin plasma concentrations and associated clinical symptoms.

Allopurinol
Interactions with allopurinol have been observed for 5-FU; with possible decreased efficacy of 5-FU. Concomitant use of allopurinol with capecitabine should be avoided.

Antivirals
Brivudine and **sorivudine MUST NOT** be prescribed with capecitabine as they may produce a life-threatening interaction. These drugs are not licensed for use in the UK but may be prescribed in other countries.

5.5 Use of Calcium and Magnesium Supplements

It is acceptable for calcium and magnesium supplements to be given concurrently with trial treatment and these are sometimes prescribed in an attempt to decrease acute neurotoxicity.

If supplements are given during the course of chemotherapy treatment this should be recorded on the Treatment CRF.

6 MANAGEMENT AND HANDLING OF INVESTIGATIONAL MEDICINAL PRODUCT (IMP)

The listed IMPs for this study are oxaliplatin, capecitabine, folinic acid and 5-fluorouracil.

All IMPs for use in this trial must be taken from existing pharmacy shelf/commercial stock. There is no provision for funding, reimbursement or discounted stock.

IMPs should be stored under the correct temperature and storage conditions as per the SmPC.

All products used in this study are licensed medications and will not be labelled specifically for the study, additional labelling containing dispensing information should be undertaken as per local practice.

IMP accountability logs will be provided for use; these must be maintained for the duration of the study and must be kept in the study pharmacy file, these will be monitored on site. Prior to March 2012, it was a requirement that copies of the completed IMP Accountability Logs also be submitted to the co-ordinating trial office when a patient had completed treatment. As of March 2012 onwards, this process was revised and the co-ordinating trial office no longer require copies of the completed IMP Accountability Logs (for either new or existing patients). Original copies of the completed IMP accountability logs should still be maintained within the study pharmacy file. At the end of the study all accountability logs should be retained with the CRFs for patients at that site as a record of drug accountability.

Patients must be asked to return any unused capecitabine tablets at each study visit; these should be counted and recorded in the accountability log, which will provide a record of compliance.

Where full L-Folinic Acid/Folinic Acid accountability cannot be recorded due to L-Folinic Acid/Folinic Acid being dispensed outside of the Pharmacy department, partial accountability (Date, Cycle No, Drug Administered, and Dose Prescribed) will be accepted.

IMP destruction, if necessary, should be undertaken in line with local policies and procedures.

7 SAFETY ASSESSMENT

Safety assessments will be performed in line with guidance specified in The Medicines for Human Use (Clinical Trials) Regulations 2004, and any subsequent amendments to it.

7.1 Definition of an Adverse Event

An adverse event is any untoward medical occurrence in a subject to whom a medicinal product has been administered, including occurrences which are not necessarily caused by or related to that product.

7.2 Adverse Event Reporting

Adverse events that are not defined as adverse reactions do not require to be recorded in the CRF (see section 7.3 for definition).

Adverse events must be recorded as they are reported, whether spontaneously volunteered or in response to questioning about wellbeing at trial visits. The questioning about adverse events will cover the current visit as well as the period of time between the previous and the current visit. All adverse events must be documented in the patient's medical records.

All adverse events must be followed until resolution, or for at least 30 days after discontinuation of study medication, whichever comes first or until toxicity has resolved to baseline or $\leq$ Grade 1, or until the toxicity is considered to be irreversible. Perceived lack of efficacy is not an adverse event.

An exacerbation of a pre-existing condition is an adverse event.

All adverse events and toxicities must be graded according to the NCI Common Terminology Criteria for adverse events (NCI-CTCAE) Version 3.0 (http://ctep.cancer.gov/reporting/ctc.html).

Abnormal laboratory test results that are deemed clinically significant by the Investigator and that lead to a change in the dosage of trial treatment or temporary or permanent discontinuation of trial treatment, or require intervention or diagnostic evaluation to assess the risk to the subject should be recorded as adverse events and instigate further investigation and follow up as appropriate.

7.3 Definition of an Adverse Reaction

An adverse reaction is any adverse event that is considered to be related to trial treatment (trial treatment is XELOX – capecitabine and oxaliplatin; and OxMdG – oxaliplatin, folinic acid and 5-FU).

7.4 Adverse Reaction Recording

All adverse reactions must be recorded in the patient's medical records. Adverse reaction data was initially recorded on the Treatment CRF, however sufficient adverse reaction data has now been collected, and the Data Monitoring and Ethics Committee (DMEC) confirmed on 13th July 2010 that this data would no longer be required to be collected on the Treatment CRF.

7.5 Definition of a Serious Adverse Event

A serious adverse event (SAE) is defined as any of the following, whether or not considered related to the trial treatment (trial treatment is XELOX - capecitabine and oxaliplatin; and OxMdG – oxaliplatin, folinic acid and 5-FU).

- Results in Death
- Life-threatening (i.e. at the time of the event)*
- Requires inpatient hospitalisation or prolongation of existing hospitalisation**
- Results in persistent or significant disability or incapacity
- Is a congenital anomaly/birth defect
- Is considered medically significant by the Investigator***

*Life threatening means that the patient was at immediate risk of death from the event as it occurred. It does not include an event that, had it occurred in a more serious form, might have caused death.

**Requires in-patient hospitalisation should be defined as a hospital admission required for treatment of an adverse event.

***Considered medically significant by the Investigator are events that may not result in death, are not life threatening, or do not require hospitalisation, but may be considered a serious adverse experience when, based upon appropriate medical judgement, the event may jeopardise the patient and may require medical or surgical intervention to prevent one of the outcomes listed above.

7.6 Definition of a Serious Adverse Reaction

A Serious Adverse Reaction (SAR) is a SAE that may be related to trial treatment. The assessment of "relatedness" is primarily the responsibility of the PI at site or agreed designee. SAEs that will be considered related will include any SAE that is documented as possibly, probably or definitely related to protocol treatment. The assessment of relatedness is made using the following:

**Relationship**	**Description**
Unrelated	There is no evidence of any causal relationship
Possible	There is some evidence to suggest a causal relationship (e.g. the event occurs within a reasonable time after administration of the trial medication). However, the influence of other factors may have contributed to this event (e.g. the patient's clinical condition, other concomitant treatments)
Probable	There is evidence to suggest a causal relationship and the influence of other factors is unlikely.
Definitely	There is clear evidence to suggest a causal relationship and other possible contributing factors can be ruled out.

7.7 Definition of Suspected Unexpected Serious Adverse Reactions (SUSARs)

A SUSAR is any suspected serious adverse reaction that is unexpected. Unexpected is any reaction that is not a known reaction listed in the Investigator Brochure or Summary of Product Characteristics of the trial treatments.

7.8 Definition of NIMPs (Non Investigational Medicinal Products)

NIMPs are "Products which are not IMPs" and are referred to in Article 2(d) of Directive 2001/20/EC and may be supplied to patients participating in a trial and used in accordance with the protocol. For instance, some clinical trial protocols require the use of medicinal products such as concomitant or rescue/escape medication for preventive, diagnostic or therapeutic reasons and/or ensure that adequate medical care is provided for the subject. They may also be used in accordance with the protocol to induce a physiological response. These medicinal products do not fall within the definition of investigational medicinal products in Directive 2001/20/EC and can be referred to as "non-investigational medicinal products" (NIMPs)."

Any SAE that could be the result of administration of a NIMP must be reported as a SAE. This can be a SAE related to the NIMP or a reaction between an IMP and NIMP.

The NIMP identified for this trial is glucose.

7.9 When SAEs are not required to be reported

SAEs that occur after consent and registration/randomisation but prior to any trial treatment do not require to be reported.

In addition, the following events do not require to be reported as SAEs:

- SAEs that are unrelated to the trial treatment.
- Hospitalisation or death due to disease progression.
- Hospitalisation for planned investigations.
- Hospitalisation for study drug administration, palliative care, terminal care or elective surgery.

The following list of known reactions to trial treatment do not require to be reported as SAEs if the patient is receiving both Oxaliplatin and Capecitabine/5FU, unless the SAE results in death. Please note if the patient is receiving Capecitabine or 5FU as single agent treatment the SAEs listed below are required to be reported as SAEs.

- Allergic reaction (including skin rash, conjunctivitis, rhinitis, anaphylactic reaction, chest pain, angioedema, hypotension, anaphylactic shock and bronchospasm).
- Anaemia, neutropenia, thrombocytopenia, leucopenia
- Insomnia, fatigue, lethargy, asthenia, rigors
- Nausea, vomiting, diarrhoea, stomatitis, mucositis, constipation, dyspepsia, intestinal obstruction, anorexia , GI reflux and dehydration
- Epistaxis
- Infection with and without neutropenia, febrile neutropenia and fungal infection
- Hypokalemia, hyper and hyponatremia, alkaline phosphatase increase, bilirubin increase, creatinine increase
- Peripheral neuropathy, sensory neuropathy
- Head ache, abdominal pain, chest pain and joint pain - myalgia and arthralgia
- Dyspnoea, cough, bronchospasm and laryngeal spasm
- Deep vein thrombosis and pulmonary embolism (these events are commonly disease related)

SAE reports will not be acknowledged or processed by the CTU for any of the above events that meet the criteria for not being reported.

7.10 Procedure for reporting SAEs

For guidance on completing the initial and follow up SAE forms please refer to the SAE Form Completion Guidelines, which will be provided by the Pharmacovigilance Office, CRUK CTU Glasgow.

If a Serious Adverse Event occurs that requires reporting, a Serious Adverse Event reporting form should be completed and faxed within 24 hours of becoming aware of the event to:

Pharmacovigilance Office, CR-UK CTU Glasgow
Fax no: +44 (0) 141 301 7213
Tel no: +44 (0) 141 301 7209/7211

The Chief Investigator will receive notification of all SAEs shortly after they are received by Pharmacovigilance and confirm agreement with the causality assessment made by the reporting Investigator.

SAEs must be reported locally by the PI at each site in accordance with the local practice at their site (i.e. Ethics Committee, R&D Office).

A follow-up report must be completed when the SAE resolves, is unlikely to change, or when additional information becomes available. If the SAE is a suspected SUSAR then follow up information must be provided as requested by the CR-UK Clinical Trials Unit and Chief Investigator.

SAEs are required to be reported for up to 30 days after last administration of study treatment. Any SAE that occurs after 30 days post treatment (with no time limit) is also required to be reported if the Investigator thinks that the SAE is related to protocol treatment (is a SAR), or is medically important. Post treatment SARs should be reported by contacting the CR-UK Clinical Trials Unit, Glasgow:

Email:	crukctupv@clinmed.gla.ac.uk
Tel:	+44(0) 141 301 7209
Fax:	+44(0) 141 301 7213

7.11 Procedure for Identifying SUSARS

The CR-UK Clinical Trials Unit will prepare a SUSAR checklist for identifying potential SUSARs. The checklist is a list of the known expected reactions to 5-FU and/or oxaliplatin and/or folinic acid and/or capecitabine against which a SAR can be checked. For any SARs not listed on the checklist the Chief Investigator will be contacted for an opinion of SUSAR status. The Chief Investigator (or designee) is responsible for deciding if a SAR is a SUSAR.

7.12 Procedure for Reporting SUSARs

The CR-UK Clinical Trials Unit is responsible for the expedited reporting of all SUSARs to the MHRA, and any other appropriate regulatory authorities, Main Research Ethics Committee, PI at trial sites and the trial Sponsor:

- Fatal or life threatening SUSARs will be reported within 7 days of the Trials Unit receiving the initial report. Any additional information will be reported within eight days of sending the first report.

- All other SUSARs will be reported within 15 days of the Trials Unit receiving the initial report.

7.14 Annual Safety Reports. Development Safety Update Reports

Annual safety reports (ASRs) will be prepared and submitted by the CR-UK Clinical Trials Unit Glasgow for all SARs reported for the trial. ASRs will be submitted to the MHRA, Main Research Ethics Committee, Sponsor and trial Investigators on the anniversary of obtaining the Clinical Trial Authorisation. After 01 September 2011 the ASR will be replaced by Development Safety Update Reports which will also be submitted on the anniversary of obtaining the Clinical Trial Authorisation.

7.15 Reference Safety Information

The Reference Safety Information (RSI) for the trial is section 4.8 undesirable effects for the SmPCs for Oxaliplatin, 5-Fluorouracil, Folinic Acid and Capecitabine. The Sponsor will identify updates to the SmPCs on an on-going basis and inform Pharmacovigilance at the CR-UK CTU of the update. The CRUK-CTU will send the updated SmPCs to the CI. The CI will be asked to confirm if the risk- benefit assessment and the clinical management of the trial participants is affected by the update to the RSI.

If the risk-benefit assessment is affected by any update to the SmPCs, then the SmPC will require regulatory approval before it can be implemented and used to assess SAE reports against expectedness to identify SUSARs. However any such updated SmPCs will be circulated to the trial sites for their information. Once regulatory approval of the updated SmPC is received it will be implemented and the RSI will change. Investigators will be notified of any RSI changes. If the risk-benefit assessment remains unchanged by the update but the clinical management of trial participants is changed then the updated RSI will not require immediate regulatory approval and will be implemented when the next DSUR reporting period ends. However the updated RSI will be circulated to the trial sites for their information. If neither the risk-benefit assessment or the clinical management of trial participants is altered by updated RSI then the updated SmPC(s) will not be implemented or circulated to sites until the current DSUR reporting period ends. Notification that the RSI has changed will given when the DSUR report is circulated to Investigators at the end of the DSUR reporting period.

8 ASSESSMENT AND FOLLOW-UP

All patients will be assessed prior to each treatment cycle (as per the schedule of assessments (on pages 8 - 9).

During follow up, CEA should be performed at each visit as per the schedule of assessments. Bloods taken for a follow up visit may be completed within one month prior to the scheduled visit. Blood tests maybe completed locally / at a patient GP. The bloods must be signed and dated and filed in the patient notes for source verification. For Years 6 – 9 follow-up visits, only CEA is required to be analysed.

The **12 week** patients have a SCOT month 4 and month 5 follow up visit (these SCOT visits are not required for the 24 week patients). The 12 week patients are then followed up at months 6, 9, 12, 18, 24, 36 and annually thereafter to trial completion.

The **24 week** patients' first SCOT follow up visit is due at month 6. The 24 week patients are then followed up at months 9, 12, 18, 24, 36 and annually thereafter to trial completion (as with the 12 week patients).
Please note the follow-up visits are scheduled in months post-randomisation date – rather than in reference to the previous visit. Please refer to the individual patient diary for specific dates.

If a patient stops their allocated treatment early or the patient experiences treatment delays the SCOT follow up visits will still be due as per the protocol schedule/patient diary, e.g. at Month 4 post randomisation date for patients allocated 12 weeks of treatment, and at Month 6 post randomisation date for patients allocated to 24 weeks of treatment. This is the required procedure even if the patient has experienced treatment delays and is still receiving chemotherapy treatment.

If for any reason a patient receives a CT scan outside the SCOT protocol, or a follow up visit is delayed or early for any given reason the patient's subsequent follow up visits should be brought back into line with the patient diary/protocol schedule as soon as possible, using the randomisation date as a bench mark.

CT scans are due for all patients (12 and 24 week patients) at the following time points:

- Month 6*
- Month 12
- Month 18*
- Month 24
- Month 36*

(*CT Scan is the preferred method of radiological assessment, however it is acceptable to use US of Liver <u>and</u> CXR combined as a substitute at the month 6, month 18 and month 36 visit only).

At Months 6, 12, 18, 24 and 36 the Follow Up CT scans should be performed as per the protocol schedule.
Please note these CT scans are to be scheduled from randomisation date - not at six monthly intervals since the last CT scan. Please refer to the individual patient diary for scheduled dates.

CT scans performed within 6 weeks (42 days) prior to a follow up visit will be acceptable for use at that SCOT follow up visit. If the time interval from any performed CT scan to a scheduled SCOT follow-up visit is greater than 42 days it is expected a separate CT scan would be completed for the scheduled SCOT follow up visit.

If a patient has reached the primary endpoint of the trial and has a recurrence, or a new primary tumour is reported (confirmed by CT scan) then the protocol schedule of CT scans and follow up bloods will no longer be required. Any post-recurrence CT scans and blood tests should be performed as per local practice at the discretion of the Investigator. All subsequent CT scan results should be recorded on the appropriate Follow Up CRF. There is also the option to complete the remainder of follow-up remotely, without the patient attending the outpatient clinic. Please see the Remote Follow-Up section below which details the change in the follow-up process if remote follow-up is to be used.

It would still be expected that the subsequent SCOT Follow Up Forms (post recurrence) are still completed at the expected time points, as per the individual patient diary.

Subsequent colonoscopy (or other surveillance of the colon) should be performed as per individual centre protocol. Investigations will be performed at other times as clinically indicated. Any relapse or incidence of new colorectal cancer must be confirmed by CT scan (and histology/cytology as appropriate).

Toxicities will be graded using the NCI-CTCAE Version 3.0 http://ctep.info.nih.gov/reporting/ctc.html

A one off update on patient status will be collected on 30th November 2018. Sites will be asked to complete a form to give the following:

- Survival data on all patients previously known to be alive
- For patients who have no record of relapse, any details of subsequent relapse

Remote Follow-Up

For patients receiving follow-up at Years 4 - 9, or for patients that have reached the primary endpoint of the trial, there is the option to complete the remainder of follow up remotely without a visit to the clinic. This can be completed by a combination of a phone call to the patient by the site research nurse, review of the patient notes and the patient having the option to complete bloods locally with a blood card. The changes need to be discussed and agreed with the patient as some patients may prefer to continue to attend the hospital clinic. If remote follow-up is preferable and agreed, the discussion and outcome should be documented in the patient's notes.

All SCOT follow-up assessments still have to be adhered to as follows:

- Patient would have the option to complete bloods locally at hospital or GP (as per schedule of assessments foot note 8).
- Blood card to be sent to the patient (please note bloods are not required post recurrence, and only CEA value is required for Years 6 - 9).
- Blood test results must be signed and dated by a member of the site trial staff and filed in the patient notes for source data verification.
- Questionnaires to be posted to the patient if the patient is participating and willing/appropriate to complete.
- Phone call to the patient for survival status and recurrence symptom information
- Review of the patient hospital notes for HEA information.
- CT scans not required.
- SCOT FU CRF to be completed with the above information as per SCOT patient diary.
- Any concerns picked up from remote FU processes should result in an OPA.
- Site needs a system in place to ensure the long term follow-up is completed by SCOT staff listed on the SCOT delegation log and Staff Contact & Responsibilities Sheet, at the required time points as per SCOT patient diary and SCOT protocol.

8.1 Quality of Life Questionnaires

At the outset of the trial sites opted to participate in the collection of quality of life and economic questionnaires (EORTC QLQ-C30 & CR29, GOG-NTX 4 and EQ-5D) at PI randomisation.

The collection of detailed toxicity (adverse reactions), QoL and economic data will only be collected for approximately 700 patients as the sample size required for these comparisons is much smaller (see Section 7.4 for full details regarding collection of adverse reaction data).

EORTC QLQ-C30 & CR29 and EQ-5D Questionnaires

These questionnaires are only completed in a sub-set of patients (patients randomised prior to 16th February 2011 from sites who opted to participate in this sub-study). For this sub-set of patients, the completion of questionnaires must continue as per the SCOT protocol. Please see the Schedule of Assessments for collection time-points.

In addition, for this sub-set of patients who have a confirmed recurrence, or evidence of new tumour, questionnaires should continue to be completed as per schedule of assessments, providing it is deemed appropriate to ask the patient to do so.

Patients participating in the Quality of Life (QoL) Study should complete the EORTC QLQ-C30 and CR29 (a colorectal module which is under development) (Appendix 6) prior to randomisation (baseline) and on day 1 of each treatment cycle (cycles 2-12). In addition, quality of life will be assessed monthly in the 12 weeks arm for the first 3 months post treatment; and in both arms of the trial at 9 and 12 months on study.

In addition to the disease specific EORTC questionnaires, the generic EQ-5D questionnaire (Appendix 8), used for the economic analysis, will require completion at the same frequency as the QoL, however, the EQ-5D questionnaire will also need to be completed by the patient at each follow up visit until study participation is complete (not to be completed by the 24 week patients at the month 6 visit). The following additional data will also be recorded for the economic analysis:

- Cytotoxic drug use at relapse
- Resource use for treating /managing adverse events
- Costs of managing cancer progression

Resource use data collection will be limited to health service resource use only in the secondary care setting.

GOG Ntx 4 Questionnaire
Similar to the QoL and EQ-5D questionnaires, these questionnaires are only completed in a sub-set of patients. Neurotoxicity is a particular concern with oxaliplatin and this will be assessed using the GOG Ntx 4 questionnaire (Appendix 7). This will require completion at the same frequency as the EQ-5D questionnaire and completed by the patient at each follow up visit until study participation is complete (not to be completed by the 24 week patients at the month 6 visit).

Completion of this questionnaire for new patients stopped on 16th February 2011. However due to insufficient data it is now requested that sites who opted into this sub-study restart the completion of the GOG Ntx 4 questionnaire in all new patients randomised to the study.

8.2 Pregnancy

Patients should agree to use reliable birth control during the time they are receiving chemotherapy and for a year after stopping chemotherapy. If the patient or their partner becomes pregnant either whilst receiving trial chemotherapy or in the first year after stopping trial chemotherapy it must be stressed that they are requested to inform their Investigator immediately. Once informed of a pregnancy, sites must immediately complete and fax a Pregnancy Notification Form to their coordinating trial office. The Pregnancy Notification Form must be updated and faxed again as soon as anything relating to the pregnancy changes such as miscarriage, termination or delivery of the baby.

9 STATISTICS AND DATA ANALYSIS

9.1 Sample Size

The study is designed as a non-inferiority trial aimed at excluding a maximum 2.5% fall in 3-year disease-free survival on the 12 week arm (from 78% on 24 week arm; this corresponds to a hazard ratio of 1.13) with 90% power at the 2.5% 1-sided level of statistical significance. Assuming the study recruits over a period of 5-years with a subsequent minimum follow-up of 2 years this will require 8,600 patients to be recruited (a 1:1 allocation will be used) and 2,750 events (relapses/deaths/new colorectal cancers) to be observed. In order to allow for losses to follow-up the actual number we will aim to recruit is 9,500.

In the MOSAIC trial the 3-year DFS on the FOLFOX arm was 78% as compared to 73% with 5 FU/LV [13]. In order to conclude non-inferiority for the 12 week arm we would wish to be confident that at least half of this benefit was retained.

It is acknowledged that disease-free survival rates in the UK may be less than those observed in the MOSAIC trial (for example 3-year disease free survival in the QUASAR study was 64%); however if this is the case this will only enhance the power of the study by increasing the number of events observed during the study period.

The number required for the toxicity, QoL and health economics comparisons is much smaller and it is proposed to collect this information only at centres who agree to participate in these aspects of the study until the required sample size is attained. A sample size of 700 patients (350 on each arm) will allow us to detect (80% power, 5% 2-sided level of statistical significance) a halving in grade 3/4 toxicity rates from 12% to 6% (12% is the rate at which grade 3/4 paraesthesia occurred in the oxaliplatin arm in the MOSAIC trial; paraesthesia was the most frequent non-haematological grade 3/4 toxicity recorded). This same sample size will allow us to detect small changes in global quality of life (assuming a difference of magnitude 7.5(29) and a standard deviation of 23(30)) with 95% power at the 1% level of statistical significance (to allow for multiple testing). This number should also allow health economics parameters to be estimated reliably, on the basis of the multivariate approach to analysis.

9.2 Analytical Plan

9.2.1 Primary efficacy analysis

The primary analysis of the DFS end-point will be based on the intention-to-treat (ITT) population with a supplementary analysis based on the per-protocol population (PP). The per-protocol population is broadly defined as those patients on the 12 week arm who receive 12 weeks of treatment (but do not exceed that) and those patients on the 24 week arm who receive more than 12 weeks treatment.

The primary analysis technique will be Cox regression which will be used to estimate the study arm effect. A test for interaction will be conducted to assess whether the duration effect depends on choice of regimen or the other clinical factors used in the minimisation. This analysis will be conducted at the end of the minimum follow-up period once the required number of events for disease-free survival have been observed.

The degree of potential bias in the PP analysis will be explored by comparing DFS, reasons for non-compliance and characteristics of the patients excluded from each arm. In the event that the PP and ITT analysis lead to differing conclusions regarding non-inferiority the reasons for this will be examined and an additional analysis conducted adjusting for non-compliance(31).

9.2.2 Secondary efficacy analysis

The secondary efficacy analysis is overall survival. This is defined as the time from the date of randomisation until death from any cause. The analysis of this end-point will use the same approach as that for disease-free survival.

9.2.3 Safety Analysis

The worst recorded toxicity grade for each patient on the NCI-CTCAE toxicity scale (version 3.0) will be summarised by treatment arm and compared using the Mann-Whitney U-test.

9.2.4 Quality of life analysis

The analysis of QoL data will be based on AUC techniques(32).

9.2.5 Comparison of randomisation time points

The method used for this will be similar to the method proposed by Carter[33], but allowing for variable recruitment rates across centres.

A recruitment method will be classified as a "success" if, on the basis of a simulation, based on the data observed in the first year, the probability of the study completing recruitment within, at most a 4-month overrun, is greater than 90%.

The final choice of method will be based on the following elements which will be reviewed by the Trials Steering Committee:-

- Whether or not the recruitment method is a "success" and the distribution of time to study completion.
- If randomisation after 12 weeks is a "success" the presumption will be that this will be the approach selected.

The final decision will also take into account the observed compliance rates and any adjustment to recruitment required to compensate for this as well as additional information available from recruitment logs.

9.2.6 Interim Analysis

The study data will be reviewed approximately annually by an independent DMEC (see section 12) from an ethical viewpoint who will address safety and efficacy issues. Conditional power methods [34] will be used to aid the committee in reaching decisions about study continuation.

The Trial Steering Committee (TSC – see section 12) will receive the report from the DMEC and crucially, after the first year of recruitment, will have the job of assessing the results of the methodological study and deciding, firstly, whether or not the recruitment rate is adequate with either method of randomisation, and if so which randomisation method is to be used for the remainder of the study.

9.2.7 Economic Analyses

The economic analysis will be presented separately for the trial period (to summarise the observed evidence in relation to cost-effectiveness) and separately for a projected lifetime cost-effectiveness of the treatment alternatives. Within trial analyses will be presented both to test the underlying hypotheses and to provide necessary parameter estimates for the lifetime cost-effectiveness model.

Quality of life utilities (from the EQ-5D) will be analysed in a similar manner to the EORTC QLQ-C30 & CR29 to establish whether these weights are different over the course of the treatment period, between the two study arms. Note that, in addition to being issued with the QLQ-C30 during treatment and follow-up during the first year, the EQ-5D will continue to be issued to patients at all subsequent follow up visits.

A multivariate analysis of quality of life using the occurrence of toxic events and the stage of any disease progression as explanatory variables will be used to inform the lifetime cost-effectiveness model [35]. The lifetime quality adjusted life-years (QALYs) will be obtained from the model and will be compared between the two arms allowing for the effects of any differential toxicity rates and any differences in the observed disease free interval and overall survival between the treatment arms.

Costs will be compared between the treatment groups for the following categories of cost: treatment cost; cost of treating toxicities and costs of treating/managing disease recurrence. The net cost difference between treatment arms will be generated from a comparison of total cost. A multivariate analysis of cost using the occurrence of toxic events and the stage of any disease progression as explanatory variables will be used to inform the lifetime cost-

effectiveness model [36]. Estimates of lifetime cost of managing the disease under the two alternative treatment strategies will be obtained from the model.

The aim of the lifetime model will be to estimate the potential cost-effectiveness of moving to a strategy of reducing the period of adjuvant therapy, together with associated uncertainty around that estimate. Probabilistic sensitivity analysis will be used to characterise uncertainty in parameters of the model driven by estimates obtained from the clinical trial [37]. In addition, structural assumptions will be tested in a series of univariate sensitivity analyses. The focus of the model will be to inform decision-making through estimation rather than hypothesis testing.

10 TRANSLATIONAL RESEARCH

Funding has been secured to support the collection of FFPE blocks and bloods for patients on the SCOT trial. The aim of collecting this material (the transSCOT study) is to establish a large biobank of colorectal cancer tissue and blood with complete and comprehensive trial quality follow-up data which will act as the foundation for many future collaborative research projects and for combined projects with other funded tissue collections. Expected research projects arising will include definition of new prognostic markers in this group of patients, including defining whether there is a sub-group of patients for whom abbreviated therapy is not appropriate; and definition of pharmacogenetic markers of 5FU/capecitabine and oxaliplatin toxicity, particularly high grade diarrhoea and neurotoxicity.

This is a hugely important and integral part of the SCOT trial that will significantly enhance the potential impact and clinical applicability of the results of the main body of the study. We are therefore keen that all researchers contribute as much as possible to this part of the study and encourage their patients to give their consent to allow this to take place.

Clearly, participation in the transSCOT study is not mandatory in order for a patient to take part in the main efficacy intervention in the SCOT trial Samples will only be collected/retrieved for those patients who consent specifically for this component of the study. Patients may consent to donate their FFPE block and / or to have their blood taken for submission to the coordinating trials office Translational Labs. It is important to note that the power of the translational study will be more greatly enhanced the larger the number of full set samples (FFPE / serum / EDTA) that we receive.

Tumour Specimens:
We plan to collect the paraffin embedded tumour tissue which will have been obtained at surgical resection of patient's primary tumour prior to entry into SCOT. Packs will be provided to send these to the CR-UK CTU Trials Office in Glasgow.

Blood samples:
We will collect blood samples – a 5ml serum sample and 2 x 9ml EDTA samples (for DNA) from each patient who consents to this. Packs will be provided for these to be sent to the OCTO Trials Office in Oxford. **If possible we would prefer it if these bloods could be taken prior to starting the adjuvant therapy**. However, it would still be useful to receive these bloods taken at any point during the treatment or follow-up period and there is still very useful translational research that we can carry out on these samples whatever the timepoint at which they are collected. Therefore please do send samples from any patient who consents to give their blood for this research.

Sample collection, storage and processing:
Further detailed instructions for the processing, labelling, handling storage and shipment of these specimens will be provided by CR-UK Clinical Trials Unit (CTU) and Oncology Clinical Trials Office (OCTO).

The custodians of all samples will be the CRUK CTU Glasgow and The University of Oxford. Proposals for specific translational research projects utilising the material will be considered by the Trial Management Group and presented to the independent Trial Steering Committee for approval.

11 REGULATORY ISSUES

11.1 Clinical Trial Authorisation (CTA)

The Cancer Research UK Clinical Trials Unit, Glasgow has received from the MHRA a Clinical Trial Authorisation (CTA) to conduct the trial and will be responsible for the maintenance of the CTA.

11.2 Ethics Approval

Ethical approval has been gained from a Main REC prior to commencement of this trial. A Site Specific Assessment (SSI) approval must have been received for each site from their local R&D Department before patients are registered at the site.

The study will be conducted in agreement with ICH GCP and the “Declaration of Helsinki” amended in Tokyo, Venice, Hong Kong (1996) and any subsequent amendments to it.

11.3 Informed Consent

Consent to enter the study must be sought from each participant only after full explanation has been given, a Patient Information Sheet (PIS) offered, and time allowed for consideration. Signed participant consent must be obtained. The Consent Forms must also be signed by the person undertaking the consent procedure at site, who <u>must</u> be detailed on the Staff Contact and Responsibilities Sheet as having this authorisation. Two original copies of the Consent Form must be signed. Any deviation from this procedure must be approved by the trial Sponsor after careful consideration of the site consent procedure. Once a patient has consented to the study a Consent Notification Form must be submitted to the responsible trials office for your site following randomisation of the patient to the trial. The right of the participant to refuse to participate without giving reasons must be respected. After the participant has entered the trial the clinician remains free to give alternative treatment to that specified in the protocol at any stage if he/she feels it is in the best interests of the participant, but the reasons for doing so must be recorded. In these cases the participants remain within the study for the purposes of follow-up and data analysis.

An original completed Consent Form must be retained at each site in the appropriate section of the Investigator File, and a photocopy placed in the patient’s case notes. All patients must be given an original of the signed Patient Information Sheet and Consent Form for their records. A Consent Notification Form must be submitted with the Registration/Randomisation Form. Consent Forms must be retained on site and not submitted to the coordinating trials office.

All participants are free to withdraw at any time from the study without giving reasons and without prejudicing further treatment or care.

If a patient wishes to withdraw their consent for use of their data, the patient should be asked to complete a Consent Withdrawal Form, and a Consent Withdrawal Notification Form should be submitted to the responsible trials unit for your site.

In the event that new or revised Patient Information Sheets/Consent Forms are required, the Sponsor will decide on a per updated Patient Information Sheet/Consent Form basis, if patients already participating in the study should be re-consented or just provided with the updated Patient Information Sheet for their information (to be noted on the PIS Distribution Log). In cases where patients need to be re-consented this should be documented on the Reconsent Log. If the PI decides that this is not in the best interests of the patient, a comment should be added to the Reconsent Log to document this decision. In the instance whereby re-consent is not required, the PI may decide it is appropriate to provide the patient with a copy of the updated Patient Information Sheet and this must be recorded on the PIS Distribution Log.

11.4 Confidentiality

National Health Service Guidelines for storage, transmittal and disclosure of patient information will be followed at all times.

This study will be carried out in accordance with ICH GCP Guidelines. Following formal admission to the study, patient data will be recorded in the hospital case record in the usual way including the circumstances of their entry to the study. Additionally data will be held in hard copy study Case Report Forms (CRFs). These files will be identified by a study number, date of birth and patient initials only.

Representatives from the Study Sponsors and from the regulatory authorities will be given access to the records that relate to the study. They will have full access to the anonymous CRFs for the purposes of data validation.

Results of the study may be communicated at scientific meetings and will contribute to the scientific literature. At no time will this be done in such a way that an individual patient may be identified.

11.5 Liability, Indemnity and Insurance

The Hospital Trust at each participating site is responsible for the following:

1. Acts and omissions of its own staff and others engaged by it, including the Clinical Trials Unit and PI;
2. Ensuring the appropriate insurance by the National Health Service Litigation Authority is in place;
3. Ensuring any non-NHS employees involved in the clinical trial have Honorary Contracts with the Trust to cover access to patients and liability arrangements.

These responsibilities are outlined and agreed within the Clinical Trial Agreement.

No special insurance is in place for patients in this study other than standard NHS liability insurance providing indemnity against clinical negligence. This does not provide cover for non-negligence e.g. harm caused by an unexpected side effect of participating in a study.

11.6 Funding

This study is being funded by a grant from the Medical Research Council (MRC) UK, however no funding is available for chemotherapy medication used in the trial and patients must be treated with existing stock at each site.

11.7 Monitoring

Central Monitoring
Study sites will be monitored centrally by checking incoming forms for compliance with the protocol, data consistency, missing data and timing. Study staff will be in regular contact with site personnel (by phone/fax/email/letter) to check on progress and deal with any queries that they may have.

On-Site Monitoring
All participating study sites will be visited by a member of the CTU/OCTO monitoring team. The PI will allow the study staff access to source documents as requested. In addition, the pharmacy department responsible for the trial will be visited to allow monitoring of the pharmacy site file and review of security and storage of trial drugs. Investigators and site staff will be notified in advance about forthcoming monitoring visits. On occasion, members of the CTU/OCTO monitoring teams may be accompanied by other trial staff from the unit for training purposes.

11.8 Audits and Inspections

The study may be subject to audit/inspection by Greater Glasgow Health Board (GGHB)/ University of Glasgow (GU) under their remit as Sponsor, the CR-UK CTU, OCTO and other regulatory bodies to ensure adherence to GCP. If an inspection is scheduled at any participating site, the site must notify the CR-UK CTU and OCTO at the earliest opportunity.

11.9 Recruitment to Other Research Studies

Clinical Trials of Investigational Medicinal Products (IMPs)
Patients must not be recruited to any trials that involve an IMP whilst they are receiving SCOT protocol treatment and during the follow up period prior to first confirmed recurrence.

Non-IMP Studies
Patients will be able to take part in questionnaire studies if they wish at any time whilst participating in the SCOT trial.

If sites wish to recruit SCOT patients to any interventional Non-IMP studies, the Sponsor and Trial Management Group will consider this on a study by study basis and where required request ethical approval to allow co-enrollment.

12 TRIAL MANAGEMENT AND DATA COLLECTION

12.1 Study Organisation

SCOT is an independent Investigator led trial run with a grant provided by the Medical Research Council (MRC) UK and sponsored by Greater Glasgow Health Board (GGHB)/University of Glasgow (GU).

Initially it was estimated that approximately 150 sites would be involved in order to enrol 9500 patients over 5 years. The study initiated in March 2008 with the anticipated completion of enrolment in March 2013, and the end of formal trial follow-up in March 2015. Following recommendation by the TSC and approval by the Sponsor and Funder, the enrolment period of the study was extended until the 29th November 2013. The study closed to recruitment on this date with a final recruitment total of 6144 patients.

The trial is being co-ordinated by two trials units, the CR-UK Clinical Trials Unit, Glasgow (CTU) and the Oncology Clinical Trials Office (OCTO), University of Oxford. Each trials unit is responsible for the management/co-ordination of approximately half of the sites participating in the trial. Sites will be informed which trials unit is responsible for their site (their coordinating trial office).

All pharmacovigilance will be managed by the CR-UK Clinical Trials Unit, Glasgow.

12.2 Study Start Up

Sites wishing to participate in the study should contact the Cancer Research UK Clinical Trials Unit (CR-UK CTU), Glasgow or the Oncology Clinical Trials Office (OCTO), University of Oxford to obtain trial information and start-up packs (containing core documents and relevant submission information/documents).

A PI must lead the study at each site, although duties may be delegated to appropriately trained members of the local trial team (as documented on the Staff Contact and Responsibilities Sheet). He/she will be responsible for providing the responsible coordinating trials office with all core documentation. Site initiation must be carried out before the site is activated – this can be done via a telephone call between the responsible trials office and the appropriate site staff or via accessing the on-line initiation slides. If the site is initiated via the on-line initiation slides, confirmation of completion of this process is requested and a short follow up call between the main contact at the site and the coordinating trial office will take place to resolve any outstanding issues/questions. The site initiation slides will be considered to be a training provision to site staff in respect of the study and also to ensure that all core information/documentation is held by both parties. The site will then be notified by e-mail/fax when they are activated on the SCOT database and are able to recruit patients to the trial. The site initiation slides can also be used to train any new staff that join the SCOT trial site team after the site initiation has taken place.

12.2.1 Core Documents

These documents consist of:

- Clinical Trial Agreement
- Site Contact Details
- Staff Contact and Responsibilities Sheet
- Trust R&D approval letter (confirming both Trust Management and SSI approvals).
- Local versions of Patient Information Sheets, Consent Forms and GP Letters and Consent Withdrawal Forms on hospital headed paper
- Up-to-date, signed and dated CVs for the Principal Investigator, Co-Investigators and Lead Pharmacist must be provided. The CV should detail the qualifications, experience and training (including GCP training) of site personnel relevant to their role in the study, and should be updated every 3 years.

If circumstances change at the site (e.g. change of PI, hospital address etc) new documents must be completed and sent with a cover letter to the trials unit responsible for the site.

12.3 Data Collection

Case Report Forms (CRFs) will be supplied electronically to sites by their coordinating trial office. These forms must be completed in accordance to the CRF completion guidelines issued with the CRFs.

Entries to the CRFs will be made in black ballpoint pen and must be legible. Any errors must be crossed out with a single stroke, the correction inserted and the change initialled and dated by

the Investigator or the appropriate site personnel with this delegated responsibility as noted on the Staff Contact and Responsibilities Sheet. Correction fluid must not be used. **Please ensure that all data submitted on CRFs are verifiable in the source documentation or that any discrepancies are recorded and explained.**

If a patient stops their allocated treatment early, the reason for this must be noted on the Treatment CRF in the 'Future Study Treatment' and 'Reasons for Early Treatment Withdrawal' sections. Patients who discontinue treatment early will still continue to be followed-up as per protocol. If the patient withdraws their consent to any further participation in the study (both treatment and follow-up) a Consent Withdrawal Form must be completed and no further follow-up is required. A Consent Withdrawal Notification Form must be submitted to the coordinating trial office.

Completed CRF pages should be sent to the appropriate trials unit for each trial site:

SCOT Study Team
Cancer Research UK Clinical Trials Unit
Flat 42 Shelley Court
Gartnavel General Hospital
Glasgow G12 0YN

or

SCOT Study Team
Oncology Clinical Trials Office (OCTO)
Department of Oncology
Old Road Campus Research Building
University of Oxford
Old Road Campus
Off Roosevelt Drive
Headington
OXFORD OX3 7DQ

Trial sites should keep a copy of all completed CRFs.

All the original CRFs must be returned to the coordinating trial office for data entry and ultimately, statistical analysis.

CRFs from the study will be stored in line with current regulatory requirements. Other essential documents, including source data, consent forms, and regulatory documentation, will be archived by, or for the Investigator, in an appropriate archive facility in line with current regulatory requirements and made available for monitoring, audit and regulatory inspection as required.

12.4 Follow-up

Patients allocated 12 weeks of treatment have a SCOT follow up visit at month 4 and month 5. For patients allocated to 24 weeks treatment, the first SCOT follow up visit is due at month 6. Both the 12 week and 24 week patients are then followed up at months 6 ,9, 12, 18, 24, 36 and annually thereafter to trial completion (please see Section 8 – Assessment and Follow Up). The maximum duration of follow-up will be 9 years of CRF capture from the date of randomisation of the first patient into the study.

A one off update on patient status will be collected on 30th November 2018. Sites will be asked to complete a form to give the following:

- Survival data on all patients previously known to be alive
- For patients who have no record of relapse, any details of subsequent relapse

Completeness of follow-up for overall survival will be enhanced in the UK by registering patients with the Medical Research Information Services (MRIS) at the NHS Information Centre for Health and Social Care, this will enable survival follow-up beyond the 9 year period.

Patients will only be flagged with MRIS if the patient has completed the relevant section of the Consent Form.

12.5 Trial Management Group (TMG)

A TMG will oversee the running of the trial. Members of the TMG will include the Chief Investigator, Co-Investigators, Project Managers, Clinical Trial Co-ordinators, Trial Statistician, IT Staff, Quality Assurance Managers and Clinical Trial Monitors.

The TMG will meet every 2 months or as required, meetings may be by teleconference.

12.6 Trial Steering Committee (TSC)

A TSC will provide overall supervision for the trial. The TSC will be responsible for monitoring the progress of the trial towards its interim and overall objectives, focusing on adherence to the protocol, Good Clinical Practice (GCP), and patient safety. The TSC will include independent members who are not directly involved in other aspects of the trial.

12.7 Data Monitoring and Ethics Committee (DMEC)

A DMEC will be established for the trial. The DMEC will assess at intervals (planned or on request) the progress of the trial, the safety data, the critical efficacy endpoints, and will make any recommendations to the Sponsor and TMG whether to continue, modify or stop the trial.

13 ALLOCATION OF STUDY RESPONSIBILITIES

The Sponsor of this clinical trial is Greater Glasgow Health Board (GGHB)/ University of Glasgow (GU).

Prior to study initiation, a Clinical Trial Agreement will be put in place between all parties (GGHB) /GU, the Cancer Research UK Clinical Trials Unit, Glasgow (CTU), Oncology Clinical Trials Office (OCTO), the Chief Investigator (CI), the PI and NHS Representative (R&D dept) at the participating site. This Agreement will fully outline each party's responsibilities in the running of the trial. In summary, they are as follows:

13.1 Sponsor Responsibilities (GGHB/GU)

The Sponsor's responsibilities will be for Authorisation and Ethics Committee opinion, GCP and Conduct, and Pharmacovigilance. The majority of the Sponsor's responsibilities have been delegated to the CTU and OCTO as the co-ordinating offices for the study. As such, the main role of the Sponsor is to ensure that the CTU and OCTO fulfil their responsibilities as outlined in the Clinical Trial Agreement and to ensure that any identified "risks" either have controls or action points put in place.

13.2 CR-UK Clinical Trials Unit (CTU) and Oncology Clinical Trials Office (OCTO)

The CTU and OCTO are responsible for the overall management of the clinical trial. This includes all regulatory submissions (ethics, R&D and CTA), all administration relating to the submissions, circulation of all correspondence to participating sites, data management, monitoring of data quality and safety, ongoing communication with participating sites, management of SAE/SUSAR reporting, and where applicable the management of any financial arrangements.

13.3 Chief Investigator (CI)

The CI has delegated the majority of his/her responsibilities to the CTU and OCTO. The CI is directly responsible for ensuring the protocol and any amendments are in place, for review of SAEs and determination if SAEs meet the criteria for a SUSAR. The CI is also responsible for

providing advice and recommendations on medical issues that arise involving the management of the patients on the study.

13.4 Participating Site

The participating site is responsible for the management of the trial at their site. This includes ensuring local R&D approval has been given, ensuring the study is conducted according to ICH GCP requirements, and ensuring the appropriate insurance or indemnity is in place. The participating site is also responsible for arranging access for on-site monitoring and auditing as identified in the study protocol and also for regulatory inspections.

13.5 Principal Investigator (PI)

The PI is responsible for the delegation of study activities at site and ensuring all personnel are adequately trained and qualified to carry out their responsibilities. Regarding the management of patients at their site, the PI is responsible for the safety and well being of trial patients, reporting any deviations from the protocol to the coordinating trial office as well as any SAEs or safety issues. Full details of the responsibilities of the PI are outlined in the Clinical Trial Agreement. Two original copies of this will be held – one with the Sponsor and the other at the participating site. A photocopy of the signed agreement will also be held at the coordinating trial office.

14 QUALITY ASSURANCE / QUALITY CONTROL

Quality Assurance/Quality Control will be maintained by the following requirements and activities:

<u>At Site</u>

- All study sites taking part in the trial will be required to participate in site initiation training to ensure compliance with the protocol and allow training on study procedures and data collection methods. This will be done via a telephone call between the responsible trials office and the appropriate site staff or via accessing the on-line initiation slides.

- Trial Investigators and site staff must ensure that the trial is conducted in compliance with the protocol, GCP and the applicable regulatory requirements.

<u>Coordinating Trial Office</u>

- The CTU and OCTO will assist the trial Investigators and check they are complying with the protocol, GCP and regulatory requirements by monitoring trial documentation. Trial data and documentation will be checked for completeness, accuracy and reliability at monitoring visits. All participating study sites will be visited by a member of the CTU or OCTO monitoring teams. Investigators and sites will be notified in advance about forthcoming monitoring visits.

<u>Centrally</u>

- Central monitoring of trial data will be performed by the trial statistician and coordinating trials office staff by checking incoming forms for compliance with the protocol, data consistency, missing data and timing.
- The CTU and OCTO will control data consistency and data quality by entering trial data onto the CTU trial database. Computerised and manual consistency checks will be performed and queries issued in cases of inconsistency or missing information. A full audit trail of any changes to the database will be maintained.

- An independent DMEC will be established to oversee the safety and interim efficacy of the trial and will report their findings and recommendations to the TSC and TMG for implementation. The complete DMEC reports will remain confidential to the DMEC members and the trial statistician.
- The TSC will ensure the trial is being managed effectively by the TMG.
- The TMG will ensure the trial is being managed according to the protocol, GCP and regulatory requirements on time and within budget.
- Non-compliance with the protocol will be discussed with the TMG and trial Sponsor. Major deviations from the protocol or significant breaches of GCP may require recruitment to be suspended temporarily at the site while an investigation of the non-compliance is conducted. The outcome of such investigations will be discussed with the trial Sponsor who will decide the appropriate course of action. The trial Sponsor will decide if recruitment can resume or if the trial requires to be terminated at the site under investigation. If there is evidence of a serious breach of GCP, the trial Sponsor may decide it is necessary to report the breach to the regulatory authorities.

15 DEFINITION OF END OF STUDY

For the purposes of the Clinical Trial Authorisation the study end date is deemed to be the date of the last visit of the last patient undergoing the trial.

For the purposes of the MREC approval, the study end date is deemed to be the date of the last visit of the last participant or the completion of any follow-up monitoring and data collection.

16 PUBLICATION POLICY AND AUTHORSHIP

The SCOT TMG is responsible for approving the content and dissemination of all publications, abstracts and presentations arising from the study and for assuring the confidentiality and integrity of the study. It will provide collaborators with approved publicity material and information updates at regular intervals during the course of the study. The definitive publications from SCOT will be written with input from the collaborators and will acknowledge all those who have contributed to the study.

No site or individual will publish data without prior approval of the TMG.

The author names on any publication will include the Chief Investigator, UK Co-Investigator(s), Study Statistician and at least one representative from the Glasgow CTU and OCTO. In addition, for participating countries who have contributed to the study recruitment, the lead investigator <u>or</u> the coordinating group collectively will be included on the authorship list. If there is any room for additional authors to be added, the top recruiting centres will be asked to nominate a co-author on behalf of their centre (the number will be dependent on the publishing journal specification).

The data arising from SCOT will belong to the trial Sponsor (GGHB) /GU). The TMG shall act as custodian of this data.

17 REFERENCES

1. Stats C. Cancer Research UK.

2. Obrand DI, Gordon PH. Incidence and patterns of recurrence following curative resection for colorectal carcinoma. Dis Colon Rectum. 1997 Jan;40(1):15-24.

3. Efficacy of adjuvant fluorouracil and folinic acid in colon cancer. International Multicentre Pooled Analysis of Colon Cancer Trials (IMPACT) investigators. Lancet. 1995 Apr 15;345(8955):939-44.

4. Haller DG CP, Macdonald JS, Mayer RJ. Fluorouracil (FU), leuconorin (LV) and levamisole (LEV) adjuvant therapy for colon cancer: five-year final report of INT-0089. . Proc Am Soc Clin Onc. 1998;1998;17:256a.

5. O'Connell MJ, Laurie JA, Kahn M, Fitzgibbons RJ, Jr., Erlichman C, Shepherd L, et al. Prospectively randomized trial of postoperative adjuvant chemotherapy in patients with high-risk colon cancer. J Clin Oncol. 1998 Jan;16(1):295-300.

6. O'Connell MJ, Mailliard JA, Kahn MJ, Macdonald JS, Haller DG, Mayer RJ, et al. Controlled trial of fluorouracil and low-dose leucovorin given for 6 months as postoperative adjuvant therapy for colon cancer. J Clin Oncol. 1997 Jan;15(1):246-50.

7. Wolmark N, Rockette H, Fisher B, Wickerham DL, Redmond C, Fisher ER, et al. The benefit of leucovorin-modulated fluorouracil as postoperative adjuvant therapy for primary colon cancer: results from National Surgical Adjuvant Breast and Bowel Project protocol C-03. J Clin Oncol. 1993 Oct;11(10):1879-87.

8. Wolmark N, Rockette H, Mamounas E, Jones J, Wieand S, Wickerham DL, et al. Clinical trial to assess the relative efficacy of fluorouracil and leucovorin, fluorouracil and levamisole, and fluorouracil, leucovorin, and levamisole in patients with Dukes' B and C carcinoma of the colon: results from National Surgical Adjuvant Breast and Bowel Project C-04. J Clin Oncol. 1999 Nov;17(11):3553-9.

9. Zaniboni A. Adjuvant chemotherapy in colorectal cancer with high-dose leucovorin and fluorouracil: impact on disease-free survival and overall survival. J Clin Oncol. 1997 Jun;15(6):2432-41.

10. Benson AB, 3rd, Schrag D, Somerfield MR, Cohen AM, Figueredo AT, Flynn PJ, et al. American Society of Clinical Oncology recommendations on adjuvant chemotherapy for stage II colon cancer. J Clin Oncol. 2004 Aug 15;22(16):3408-19.

11. Figueredo A, Charette ML, Maroun J, Brouwers MC, Zuraw L. Adjuvant therapy for stage II colon cancer: a systematic review from the Cancer Care Ontario Program in evidence-based care's gastrointestinal cancer disease site group. J Clin Oncol. 2004 Aug 15;22(16):3395-407.

12. Gray RG BJ, Hills R et al. QUASAR: a randomised stuy of adjuvant chemotherapy versus observation including 3238 colorectal cancer patients. Proc Am Soc Clin Oncol 2004;23(246):Abstract #3501.

13. Andre T, Boni C, Mounedji-Boudiaf L, Navarro M, Tabernero J, Hickish T, et al. Oxaliplatin, fluorouracil, and leucovorin as adjuvant treatment for colon cancer. N Engl J Med. 2004 Jun 3;350(23):2343-51.

14. Twelves C, Wong A, Nowacki MP, Abt M, Burris H, 3rd, Carrato A, et al. Capecitabine as adjuvant treatment for stage III colon cancer. N Engl J Med. 2005 Jun 30;352(26):2696-704.

15. Scheithauer W, McKendrick J, Begbie S, Borner M, Burns WI, Burris HA, et al. Oral capecitabine as an alternative to i.v. 5-fluorouracil-based adjuvant therapy for colon cancer: safety results of a randomized, phase III trial. Ann Oncol. 2003 Dec;14(12):1735-43.

16. Kuebler JP, Wieand HS, O'Connell MJ, Smith RE, Colangelo LH, Yothers G, et al. Oxaliplatin combined with weekly bolus fluorouracil and leucovorin as surgical adjuvant

chemotherapy for stage II and III colon cancer: results from NSABP C-07. J Clin Oncol. 2007 Jun 1;25(16):2198-204.

17. Saltz LB, Niedzwiecki D, Hollis D, Goldberg RM, Hantel A, Thomas JP, et al. Irinotecan fluorouracil plus leucovorin is not superior to fluorouracil plus leucovorin alone as adjuvant treatment for stage III colon cancer: results of CALGB 89803. J Clin Oncol. 2007 Aug 10;25(23):3456-61.

18. Van Cutsem E LR, et al. Randomised phase III trial comparing infused irinotecan/5-fluorouracil (5-FU)/folinic acid (IF) versus 5-FU/FA (F) in stage III colon cancer patients (PETACC3). Proc Am Soc Clin Onc 2005;LBA8.

19. Ychou M RJ, Douillard R, et al A phase III randomised trial of LV5FU2 + CPT11 vs LV5FU2 alone in adjuvant high risk colon cancer (FNCLCC Accord02/FFCD9802). Proc Am Soc Clin Onc. 2005;Abstract 3502.

20. Hurwitz H, Fehrenbacher L, Novotny W, Cartwright T, Hainsworth J, Heim W, et al. Bevacizumab plus irinotecan, fluorouracil, and leucovorin for metastatic colorectal cancer. N Engl J Med. 2004 Jun 3;350(23):2335-42.

21. Cunningham D, Humblet Y, Siena S, Khayat D, Bleiberg H, Santoro A, et al. Cetuximab monotherapy and cetuximab plus irinotecan in irinotecan-refractory metastatic colorectal cancer. N Engl J Med. 2004 Jul 22;351(4):337-45.

22. Extra JM, Espie M, Calvo F, Ferme C, Mignot L, Marty M. Phase I study of oxaliplatin in patients with advanced cancer. Cancer Chemother Pharmacol. 1990;25(4):299-303.

23. Ashley AC, Sargent DJ, Alberts SR, Grothey A, Campbell ME, Morton RF, et al. Updated efficacy and toxicity analysis of irinotecan and oxaliplatin (IROX) : intergroup trial N9741 in first-line treatment of metastatic colorectal cancer. Cancer. 2007 Aug 1;110(3):670-7.

24. Schrag D. The price tag on progress--chemotherapy for colorectal cancer. N Engl J Med. 2004 Jul 22;351(4):317-9.

25. Aballea S, Chancellor JV, Raikou M, Drummond MF, Weinstein MC, Jourdan S, et al. Cost-effectiveness analysis of oxaliplatin compared with 5-fluorouracil/leucovorin in adjuvant treatment of stage III colon cancer in the US. Cancer. 2007 Mar 15;109(6):1082-9.

26. Cassidy J, Douillard JY, Twelves C, McKendrick JJ, Scheithauer W, Bustova I, et al. Pharmacoeconomic analysis of adjuvant oral capecitabine vs intravenous 5-FU/LV in Dukes' C colon cancer: the X-ACT trial. Br J Cancer. 2006 Apr 24;94(8):1122-9.

27. Saini A, Norman AR, Cunningham D, Chau I, Hill M, Tait D, et al. Twelve weeks of protracted venous infusion of fluorouracil (5-FU) is as effective as 6 months of bolus 5-FU and folinic acid as adjuvant treatment in colorectal cancer. Br J Cancer. 2003 Jun 16;88(12):1859-65.

28. Smith IE, O'Brien MER, Talbot DC, Nicolson MC, Mansi LJ, Hickish TF, Norton A, Ashley S. Duration of chemotherapy in advanced non small cell lung cancer: a randomised trial of 3 versus 6 courses of Mitomycin, Vinblastine and Cisplatin. J Clin Oncol 2001 19:1336-1343.

29. Osoba D, Rodrigues G, Myles J, Zee B, Pater J. Interpreting the significance of changes in health-related quality-of-life scores. J Clin Oncol. 1998 Jan;16(1):139-44.

30. Aaronson NK, Ahmedzai S, Bergman B, Bullinger M, Cull A, Duez NJ, et al. The European Organization for Research and Treatment of Cancer QLQ-C30: a quality-of-life instrument for use in international clinical trials in oncology. J Natl Cancer Inst. 1993 Mar 3;85(5):365-76.

31. Cuzick J, Edwards R, Segnan N. Adjusting for non-compliance and contamination in randomized clinical trials. Stat Med. 1997 May 15;16(9):1017-29.

32. Qian W, Parmar MK, Sambrook RJ, Fayers PM, Girling DJ, Stephens RJ. Analysis of messy longitudinal data from a randomized clinical trial. MRC Lung Cancer Working Party. Stat Med. 2000 Oct 15;19(19):2657-74.

33. Carter RE. Application of stochastic processes to participant recruitment in clinical trials. Control Clin Trials. 2004 Oct;25(5):429-36.

34. DeMets DL Futility approaches to interim monitoring committees - *Clinical Trials* 2006; 3: 522-529.

35. Clarke P, Gray A, Holman R. Estimating utility values for health states of type 2 diabetic patients using the EQ-5D (UKPDS 62). Med Decis Making. 2002 Jul-Aug;22(4):340-9.

36. Clarke P, Gray A, Legood R, Briggs A, Holman R. The impact of diabetes-related complications on healthcare costs: results from the United Kingdom Prospective Diabetes Study (UKPDS Study No. 65). Diabet Med. 2003 Jun;20(6):442-50.

37. Briggs A. Statistical methods for cost-effectiveness analysis alongside clinical trials. Contributed chapter in Jones AM (Ed) Elgar Companion to Health Economics 2006, Elgar: London. 2006.

APPENDIX 1: WHO PERFORMANCE STATUS

0	Able to carry out all normal activity without restriction
1	Restricted in physically strenuous activity but ambulatory and able to carry out light work
2	Ambulatory and capable of all self-care but unable to carry out any work: up and about more than 50% of waking hours
3	Capable only of limited self-care; confined to bed or chair more than 50% of waking hours
4	Completely disabled; cannot carry out any self-care; totally confined to bed or chair

APPENDIX 2: COCKCROFT-GAULT EQUATION

$$\textbf{Male} = \frac{1.23 \times (140 - \text{age}) \times \text{weight (kg)}}{\text{Serum Creatinine } (\mu\text{mol/l})}$$

$$\textbf{Female} = \frac{1.05 \times (140 - \text{age}) \times \text{weight (kg)}}{\text{Serum Creatinine } (\mu\text{mol/l})}$$

APPENDIX 3: CAPECITABINE DOSE BANDING

The maximum body surface area for the treatment of patients in this study is 2.2m². If the calculated body surface area is greater, then the treatment should be rounded down to 2.2m².

This appendix is a guideline for the dose banding of capecitabine for this study. Where possible, sites should dose band according to the following tables:
(Where required, it is acceptable for sites to use their own dose banding tables to extend BSA to 2.2m^2)

Number of capecitabine 500mg tablets to be prescribed and dispensed based on patient's body surface area and required dose (capping the body surface area as above).

Table 1: Capecitabine starting dose of 1000mg/m² b.d.

For patients under 70 years old with good renal function and, at the Investigator's discretion, for patients ≥ 70 years old with good renal function.

Patient's BSA m²	1000mg/m² b.d.		Total dose (mg/day)	Total no. tabs (14 days)
	am dose	pm dose		
≤ 1.12	2	2	2000	56
1.13 – 1.36	2	3	2500	70
1.37 – 1.62	3	3	3000	84
1.63 – 1.86	3	4	3500	98
≥ 1.87	4	4	4000	112

Table 2: Capecitabine dose reduction of 25% for patients starting at 1000mg/m² b.d. = 750mg/m² b.d.

For patients with impaired renal function, i.e. creatinine clearance 30-50ml/min and for patients ≥ 70 years old who are not deemed fit for full dose as per Investigator discretion.

Patient's BSA m²	25% dose reduction to 750mg/m² b.d.		Total dose (mg/day)	Total no. tabs (14 days)
	am dose	pm dose		
≤ 1.14	1	2	1500	42
1.15 – 1.49	2	2	2000	56
1.50 – 1.84	2	3	2500	70
≥ 1.85	3	3	3000	84

Table 3: Capecitabine dose reduction of 50% for patients starting at 1000mg/m² b.d. = 500mg/m² b.d.

Patient's BSA m²	50% dose reduction to 500mg/m² b.d.		Total dose (mg/day)	Total no. tabs (14 days)
	am dose	pm dose		
≤ 1.24	1	1	1000	28
1.25 – 1.74	1	2	1500	42
≥ 1.75	2	2	2000	56

APPENDIX 4: NEW YORK HEART ASSOCIATION STAGING (NYHA)

- **Class 1:** Subjects with no limitation of activities; they suffer no symptoms from ordinary activities.
- **Class 2:** Subjects with slight, mild limitation of activity; they are comfortable with rest or mild exertion.
- **Class 3:** Subjects with marked limitation of activity; they are comfortable only at rest.
- **Class 4:** Subjects who should be at complete rest, confined to a bed or chair, any physical activity brings on discomfort and symptoms occur at rest.

APPENDIX 5: EXPECTED TOXICITY PROFILE OF STUDY MEDICATIONS

Toxicity of 5-FU

**Common**	Neutropenia<br>Anaemia<br>Thrombocytopenia<br>Stomatitis and mouth ulcers<br>Diarrhoea<br>Anorexia<br>Changes in taste<br>Watery eyes or sensitivity to sunlight<br>Venous tracking
**Less common**	Hand-foot syndrome<br>Discoloration of the skin<br>Rash or itching<br>Skin sensitivity to sunlight<br>Hair loss<br>Discoloration of nails, loss of nails<br>Cracking, peeling or excessively dry skin

Toxicity of Capecitabine

**Common**	Fatigue<br>Diarrhoea<br>Hand -foot syndrome<br>Nausea and vomiting<br>Skin reactions (increased pigmentation, itching, dry skin)<br>Abnormalities in liver function tests.<br>Anaemia<br>Lymphopenia
**Less common**	Neutropenia<br>Thrombocytopenia<br>Abdominal pain<br>Anorexia<br>Stomatitis and mouth ulcers<br>Numbness or tingling of hands and/or feet (usually associated with hand-foot syndrome)<br>Swelling of the ankles and/or feet<br>Fever<br>Constipation<br>Eye irritation<br>Headache<br>Joint and muscle pain

Toxicity of Oxaliplatin

**Common**	Numbness or tingling of the hands or feet - *this condition may be exacerbated by exposure to the cold*<br>Nausea and vomiting<br>Diarrhoea<br>Fatigue<br>Anaemia<br>Thrombocytopenia<br>Constipation<br>Changes in liver function tests, liver damage
**Less common**	Neutropenia<br>Fever<br>Headache<br>Insomnia<br>Stomatitis and mouth ulcers<br>Anorexia<br>Abdominal pain<br>Back pain<br>Abnormalities in renal function tests

APPENDIX 6: EORTC QLQ-C30 & CR29 (COLORECTAL MODULE)

We are interested in some things about you and your health. Please answer all of the questions yourself by circling the number that best applies to you. There are no "right" or "wrong" answers. The information that you provide will remain strictly confidential.

Please fill in your initials: _______________

Your birth date (Day, Month, Year): _____/_____/____

Today's date (Day, Month, Year): _____/_____/____

		Not at All	A Little	Quite a Bit	Very Much
1.	Do you have any trouble doing strenuous activities, like carrying a heavy shopping bag or a suitcase?	1	2	3	4
2.	Do you have any trouble taking a <u>long</u> walk?	1	2	3	4
3.	Do you have any trouble taking a <u>short</u> walk outside of the house?	1	2	3	4
4.	Do you need to stay in bed or a chair during the Day?	1	2	3	4
5.	Do you need help with eating, dressing, washing yourself or using the toilet?	1	2	3	4

**During the past week:**		Not at All	A Little	Quite a Bit	Very Much
6.	Were you limited in doing either your work or other daily activities?	1	2	3	4
7.	Were you limited in pursuing your hobbies or other leisure time activities?	1	2	3	4
8.	Were you short of breath?	1	2	3	4
9.	Have you had pain?	1	2	3	4
10.	Did you need to rest?	1	2	3	4
11.	Have you had trouble sleeping?	1	2	3	4
12.	Have you felt weak?	1	2	3	4
13.	Have you lacked appetite?	1	2	3	4

During the past week:		Not at All	A Little	Quite a Bit	Very Much
14.	Have you felt nauseated?	1	2	3	4
15.	Have you vomited?	1	2	3	4
16.	Have you been constipated?	1	2	3	4
17.	Have you had diarrhoea?	1	2	3	4
18.	Were you tired?	1	2	3	4
19.	Did pain interfere with your daily activities?	1	2	3	4
20.	Have you had difficulty in concentrating on things, like reading a newspaper or watching television?	1	2	3	4
21.	Did you feel tense?	1	2	3	4
22.	Did you worry?	1	2	3	4
23.	Did you feel irritable?	1	2	3	4
24.	Did you feel depressed?	1	2	3	4
25.	Have you had difficulty remembering things?	1	2	3	4
26.	Has your physical condition or medical treatment interfered with your family life?	1	2	3	4
27.	Has your physical condition or medical treatment interfered with your social activities?	1	2	3	4
28.	Has your physical condition or medical treatment caused you financial difficulties?	1	2	3	4

For the following questions please circle the number between 1 and 7 that best applies to you

29. How would you rate your overall health during the past week?

1 2 3 4 5 6 7

Very poor Excellent

30. How would you rate your overall quality of life during the past week?

1 2 3 4 5 6 7

Very poor Excellent

Please go on to the next page

©Copyright 1995 EORTC Study Group on Quality of Life. All rights reserved.
Version 3.0

CR29 (colorectal module)

Patients sometimes report that they have the following symptoms or problems. Please indicate the extent to which you have experienced these symptoms or problems during the past week. Please answer by circling the number that best applies to you.

During the past week:	Not at All	A Little	Quite a Bit	Very Much
31. Did you urinate frequently during the day?	1	2	3	4
32. Did you urinate frequently during the night?	1	2	3	4
33. Have you had any unintentional release (leakage) of urine?	1	2	3	4
34. Did you have pain when you urinated?	1	2	3	4
35. Did you have abdominal pain?	1	2	3	4
36. Did you have pain in your buttocks/anal area/rectum?	1	2	3	4
37. Did you have a bloated feeling in your abdomen?	1	2	3	4
38. Have you blood in your stools?	1	2	3	4
39. Have you had mucus in your stools?	1	2	3	4

During the past week:	Not at All	A Little	Quite a Bit	Very Much
40. Did you have a dry mouth?	1	2	3	4
41. Have you lost hair as a result of your treatment?	1	2	3	4
42. Have you had problems with your sense of taste?	1	2	3	4
43. Were you worried about your health in the future?	1	2	3	4
44. Have you worried about your weight?	1	2	3	4
45. Have you felt physically less attractive as a result of your disease or treatment?	1	2	3	4
46. Have you been feeling less feminine/masculine as a result of your disease or treatment?	1	2	3	4
47. Have you been dissatisfied with your body?	1	2	3	4

48. Do you have a stoma bag (colostomy/ileostomy)?
(please circle the correct answer) Yes No

Please go on to the next page

During the past week: Not at A Quite Very All Little a Bit Much

Answer these questions ONLY IF YOU HAVE A STOMA BAG, if not please continue below:

During the past week:	Not at All	A Little	Quite a Bit	Very Much
49. Have you had unintentional release of gas/flatulence from your stoma bag?	1	2	3	4
50. Have you had leakage of stools from your stoma bag?	1	2	3	4
51. Have you had sore skin around your stoma?	1	2	3	4
52. Did frequent bag changes occur during the day?	1	2	3	4
53. Did frequent bag changes occur during the night?	1	2	3	4
54. Did you feel embarrassed because of your stoma?	1	2	3	4
55. Did you have problems caring for your stoma?	1	2	3	4

Answer these questions ONLY IF YOU DO NOT HAVE A STOMA BAG:

49. Have you had unintentional release of gas/flatulence from your back passage?	1	2	3	4
50. Have you had leakage of stools from your back passage?	1	2	3	4
51. Have you had sore skin around your anal area?	1	2	3	4
52. Did frequent bowel movements occur during the day?	1	2	3	4
53. Did frequent bowel movements occur during the night?	1	2	3	4
54. Did you feel embarrassed because of your bowel movement?	1	2	3	4

During the past 4 weeks:	Not at All	A Little	Quite a Bit	Very Much
**For men only:**				
56. To what extent were you interested in sex?	1	2	3	4
57. Did you have difficulty getting or maintaining an erection?	1	2	3	4
**For women only:**				
58. To what extent were you interested in sex?	1	2	3	4
59. Did you have pain or discomfort during intercourse?	1	2	3	4

© QLQCR29 Copyright 2006 EORTC Quality of Life Group. All rights reserved. (phase III module). Version 2.1

APPENDIX 7: GOG NTX 4 QUESTIONNAIRE

Please note that questionnaire **must** be completed by the **patient**.

By circling one (1) number per line, please indicate how true each statement has been for you during the past 7 days.

		Not at all	A little bit	Some what	Quite a bit	Very much
NTX 1	I have numbness or tingling in my hands	0	1	2	3	4
NTX 2	I have numbness or tingling in my feet	0	1	2	3	4
NTX 3	I feel discomfort in my hands	0	1	2	3	4
NTX 4	I feel discomfort in my feet	0	1	2	3	4

US English – Copyright 1987, 1997

APPENDIX 8: EQ-5D QUESTIONNAIRE

Please note that questionnaire **must** be completed by the **patient**.

By placing a tick in one box in each group below, please indicate which statements best describe your own health state today.

Mobility

I have no problems in walking about ❑
I have some problems in walking about ❑
I am confined to bed ❑

Self Care

I have no problems with self-care ❑
I have some problems washing or dressing myself ❑
I am unable to wash or dress myself ❑

Usual Activities*(e.g. work, study, housework, family or leisure activities)*

I have no problems with performing my usual activities ❑
I have some problems with performing my usual activities ❑
I am unable to perform my usual activities ❑

Pain / Discomfort

I have no pain or discomfort ❑
I have moderate pain or discomfort ❑
I have extreme pain or discomfort ❑

Anxiety / Depression

I am not anxious or depressed ❑
I am moderately anxious or depressed ❑
I am extremely anxious or depressed ❑

To help people say how good or bad a health state is, we have drawn a scale (rather like a thermometer) on which the best state you can imagine is marked 100 and the worst state you can imagine is marked 0.

We would like you to indicate on this scale how good or bad your own health is today, in your opinion. Please do this by drawing a line from the box below to whichever point on the scale indicates how good or bad your health state is today.

Your own health state today

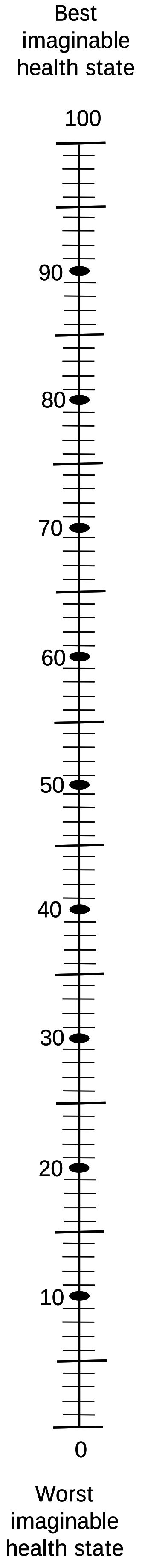

APPENDIX 9: DECLARATION OF HELSINKI

DECLARATION OF HELSINKI
WORLD MEDICAL ASSOCIATION DECLARATION OF HELSINKI
Ethical Principles for Medical Research Involving Human Subjects

Adopted by the 18th WMA General Assembly Helsinki, Finland, June 1964 and amended by the 29th WMA General Assembly, Tokyo, Japan, October 1975 35th WMA General Assembly, Venice, Italy, October 1983 41st WMA General Assembly, Hong Kong, September 1989 48th WMA General Assembly, Somerset West, Republic of South Africa, October 1996

INTRODUCTION

It is the mission of the physician to safeguard the health of the people. His or her knowledge and conscience are dedicated the fulfilment of this mission. The Declaration of Geneva of the World Medical Assembly binds the physician with the words, "The health of my patient will be my first consideration," and the International Code of Medical Ethics declares that, "A physician shall act only in the patient's interest when providing medical care which might have the effect of weakening the physical and mental condition of the patient."

The purpose of biomedical research involving human subjects must be to improve diagnostic, therapeutic and prophylactic procedures and the understanding of the aetiology and pathogenesis of disease.

In current medical practice most diagnostic, therapeutic or prophylactic procedures involve hazards. This applies especially to biomedical research. Medical progress is based on research which ultimately must rest in part on experimentation involving human subjects.

In the field of biomedical research a fundamental distinction must be recognized between medical research in which the aim is essentially diagnostic or therapeutic for a patient, and medical research, the essential object of which is purely scientific and without implying direct diagnostic or therapeutic value to the person subjected to the research.

Special caution must be exercised in the conduct of research which may affect the environment, and the welfare of animals used for research must be respected.

Because it is essential that the results of laboratory experiments be applied to human beings to further scientific knowledge and to help suffering humanity, the World Medical Association has prepared the following recommendations as a guide to every physician in biomedical research involving human subjects. They should be kept under review in the future. It must be stressed that the standards as drafted are only a guide to physicians all over the world. Physicians are not relieved from criminal, civic and ethical responsibilities under the laws of their own countries.

I. Basic Principles

1 Biomedical research involving human subjects must conform to generally accepted scientific principles and should be based on adequately performed laboratory and animal experimentation and on a thorough knowledge of the scientific literature.

2. The design and performance of each experimental procedure involving human subjects should be clearly formulated in an experimental protocol which should be transmitted for consideration, comment and guidance to a specially appointed committee independent of the investigator and the sponsor provided that this independent committee is in conformity with the laws and regulations of the country in which the research experiment is performed.

3. Biomedical research involving human subjects should be conducted only by scientifically qualified persons and under the supervision of a clinically competent medical person. The responsibility for the human subject must always rest with a medically qualified person and never rest on the subject of the research, even though the subject has given his or her consent.

4. Biomedical research involving human subjects cannot legitimately be carried out unless the importance of the objective is in proportion to the inherent risk to the subject.

5. Every biomedical research involving human subjects should be preceded by careful assessment of predictable risks in comparison with foreseeable benefits to the subject or to others. Concern for the interest of the subject must always prevail over the interests of science and society.

6. The right of the research subject to safeguard his or her integrity must always be respected. Every precaution should be taken to respect the privacy of the subject and to minimize the impact of the study on the subject's physical and mental integrity and on the personality of the subject.

7. Physicians should abstain from engaging in research projects involving human subjects unless they are satisfied that the hazards involved are believed to be predictable. Physicians should cease any investigation if the hazards are found to outweigh the potential benefits.

8 In publication of the results of his or her research, the physician is obliged to preserve the accuracy of the results. Reports of experimentation not in accordance with the principles laid down in this Declaration should not be accepted for publication.

9. In any research on human beings, each potential subject must be adequately informed of the aims, methods, anticipated benefits and potential hazards of the study and the discomfort it may entail. He or she should be informed that he or she is a liberty to abstain from participation in the study and that he or she is free to withdraw his or her consent to participation at any time. The physician should then obtain the subject's freely-given informed consent, preferably in writing.

10. When obtaining informed consent for the research project the physician should be particularly cautious if the subject is in a dependent relationship to him or her or may consent under duress. In that case the informed consent should be obtained by a physician who is not engaged in the investigation and who is completely independent of this official relationship.

11. In case of legal incompetence, informed consent should be obtained from the legal guardian in accordance with national legislation. Where physical or mental incapacity makes it impossible to obtain informed consent, or when the subject is a minor, permission from the responsible relative replaces that of the subject in accordance with national legislation.

Whenever the minor child is in fact able to give consent, the minor's consent must be obtained in addition to the consent of the minor's legal guardian.

12. The research protocol should always contain a statement of the ethical considerations involved and should indicate that the principles enunciated in the present Declaration are complied with.

II. Medical research combined with clinical care (Clinical research)

1. In the treatment of the sick person, the physician must be free to use a new diagnostic and therapeutic measure, if in his or her judgement it offers hope of saving life, reestablishing health or alleviating suffering.

2. The potential benefits, hazards and discomfort of a new method should be weighed

against the advantages of the best current diagnostic and therapeutic methods.

3. In any medical study, every patient --including those of a control group, if any -- should be assured of the best proven diagnostic and therapeutic method. This does not exclude the use of inert placebo in studies where no proven diagnostic or therapeutic method exists.

4. The refusal of the patient to participate in a study must never interfere with the physician-patient relationship.

5. If the physician considers it essential not to obtain informed consent, the specific reasons for this proposal should be stated in the experimental protocol for transmission to the independent committee (I,2).

6. The physician can combine medical research with professional care, the objective being the acquisition of new medical knowledge, only to the extent that medical research is justified by its potential diagnostic or therapeutic value for the patient.

III. Non-therapeutic biomedical research involving human subjects (Non-clinical biomedical research)

1. In the purely scientific application of medical research carried out on a human being, it is the duty of the physician to remain the protector of the life and health of that person on whom biomedical research is being carried out.

2. The subject should be volunteers -either healthy persons or patients for whom the experimental design is not related to the patient's illness.

3. The investigator or the investigating team should discontinue the research if in his/her or their judgement it may, if continued, be harmful to the individual.

4. In research on man, the interest of science and society should never take precedence over considerations related to the well being of the subject.

APPENDIX 10: PATIENT WITHDRAWAL QUESTIONNAIRE

CONFIDENTIAL

We are interested in learning more about why patients leave clinical trials or stop trial treatment early and would be very grateful if you could complete this questionnaire about why you are leaving the SCOT study, or stopping your SCOT treatment early.

Today's Date: _ _ /_ _ / _ _

Please tick **ALL** that apply:

**I am leaving the SCOT study/stopping my trial treatment early because……**	**Tick box if this contributed to your reason for leaving the trial or stopping trial treatment early**
1. My illness has got worse	
2. I was having bad side-effects	
3. I was not happy with the idea of randomisation	
4. I wanted the doctor to choose the length of my treatment rather than the computer	
5. I had too much information about the study	
6. I did not have enough information about the study	
7. Taking part in the trial was not what I expected	
8. I wanted my chemotherapy to continue for longer	
9. The doctor advised me to leave the trial/stop the trial treatment	
10. My family wanted me to leave the trial/stop the trial treatment	
11. I was spending too much time at the hospital	
12. I wanted to know at the start of the trial how many months I would be getting chemotherapy	
13. Difficulty with carer / parental obligations	
14. Transport problems	
15. Financial reasons	
16. Other reason(s) – please say what…..	

From the numbered statements above, what is the **MOST IMPORTANT** reason for you leaving the trial or stopping the trial treatment early?	Please write answer here<br>Number

Thank you very much for taking the time to complete this questionnaire.

APPENDIX 11: SCOT TUMOUR STAGING GUIDELINE

TNM staging		Dukes' Stage	Eligible for SCOT?
Tis, N0, M0	Stage O		No
T1, N0, M0<br>T2, N0, M0	Stage I	A	No
**T3, N0, M0**	**Stage II A**	**B**	**Yes**, but only if have one or more of the following high risk factors:<br>▪ lymphatic invasion<br>▪ vascular invasion<br>▪ perineural invasion<br>▪ peritoneal involvement<br>▪ poor differentiation<br>▪ obstruction and/or perforation of the primary tumour during the pre-operative period<br>▪ less than 10 nodes examined
**T4, N0, M0**	**Stage II B**	**B**	**Yes**
**T1, N1, M0<br>T2, N1, M0**	**Stage III A**	**C**	**Yes**
**T3, N1, M0**	**Stage III B**	**C**	**Yes**
**T4, N1, M0**	**Stage III B**	**C**	**Yes**
**T1, N2, M0<br>T2, N2, M0<br>T3, N2, M0**	**Stage III C**	**C**	**Yes**
**T4, N2, M0**	**Stage III C**	**C**	**Yes**
Any T, Any N, M1	Stage IV		No
